# A Biomimetic Nanoparticle System Intercepts and Degrades Thrombospondin‐1 to Restore Vascular Homeostasis After Ischemic Injury

**DOI:** 10.1002/advs.75344

**Published:** 2026-04-20

**Authors:** Haorui Wang, Xiandi Meng, Yanbao Xin, Kuirong Mao, Huizhu Tan, Changkai Ma, Xiuxiu Cong, Mengfei Zhao, Meiling Yu, Si Chen, Yue Hou, Yong‐Guang Yang, Tianmeng Sun

**Affiliations:** ^1^ Key Laboratory of Organ Regeneration and Transplantation of Ministry of Education The First Hospital Jilin University Changchun Jilin China; ^2^ National‐local Joint Engineering Laboratory of Animal Models For Human Diseases Changchun Jilin China; ^3^ International Center of Future Science Jilin University Changchun Jilin China; ^4^ State Key Laboratory of Kidney Diseases Beijing China; ^5^ State Key Laboratory of Supramolecular Structure and Materials Jilin University Changchun Jilin China

**Keywords:** ischemia‐reperfusion injury (IRI), liver inflammation, liver sinusoidal endothelial cells, PLGA nanoparticles, thrombospondin‐1‐CD47

## Abstract

The thrombospondin‐1 (TSP‐1)/CD47 signaling axis plays a critical role in endothelial injury during hepatic ischemia‐reperfusion (IR). Targeting this pathway represents a promising strategy to prevent liver IR injury; however, existing CD47 antagonists cause severe hematologic side effects due to nonspecific binding to erythrocytes. This study developed TAX2 (CD47‐derived peptide with the sequence CEVSQLLKGDAC)‐functionalized biodegradable nanoparticles (TAX2‐NPs) that specifically capture TSP‐1 and block its interaction with CD47 on liver sinusoidal endothelial cells (LSECs). TAX2‐NPs are composed of PLGA and DSPE‐PEG‐Mal and are conjugated with the cyclic peptide TAX2, derived from the TSP‐1‐binding domain of CD47. Upon systemic administration, TAX2‐NPs preferentially accumulate in the injured liver, where they competitively bind TSP‐1, preventing endothelial apoptosis and preserving sinusoidal integrity. Captured TSP‐1 is subsequently internalized by macrophages and degraded via the autophagy‐lysosome pathway, thereby eliminating residual extracellular TSP‐1 and mitigating its prolonged pathogenic effects. Collectively, this study presents a ligand‐targeted nanotherapeutic strategy that integrates TSP‐1 sequestration, signaling blockade, and autophagy‐mediated degradation, offering a safe and effective approach to protect the liver from ischemia‐reperfusion injury.

## Graphical abstract

TAX2‐NPs capture extracellular TSP‐1 in the injured liver and promote its macrophage‐mediated autophagic degradation. This process blocks TSP‐1/CD47 signaling, restores VEGFR2‐AKT activity, preserves endothelial function, and mitigates hepatic ischemia‐reperfusion injury.

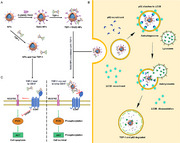

## 1 Introduction

Liver IRI is a major cause of hepatic dysfunction following hepatectomy and transplantation, leading to postoperative complications and graft failure [1, 2, 3, 4]. LSECs are the first and most sensitive to ischemic insult [5, 6]. During ischemia, LSECs experience hypoxia‐induced metabolic stress, resulting in cytoskeletal disruption [7]. Upon reperfusion, the sudden influx of oxygen triggers reactive oxygen species (ROS) production and inflammatory cytokine release, which amplify endothelial injury, increase vascular permeability, and promote thrombosis and tissue edema [8, 9]. Protecting LSEC integrity is therefore critical for mitigating IRI and preserving hepatic microcirculatory function.

TSP‐1, a large trimeric matricellular glycoprotein, has emerged as a key mediator of endothelial dysfunction during IRI [10, 11]. TSP‐1 is transiently upregulated in damaged tissues and secreted by macrophages [12], endothelial cells [13], and other parenchymal cells [14] in response to stress signals such as hypoxia and inflammation. Through its C‐terminal domain, TSP‐1 binds to CD47 on the endothelial surface, suppressing nitric oxide (NO)‐cGMP signaling, inhibiting eNOS activation [15], and stimulating NADPH oxidase‐derived ROS generation [16]. These effects collectively induce vasoconstriction, impair microcirculatory perfusion, and propagate ischemic injury. Targeting the TSP‐1/CD47 interaction, therefore, represents a rational strategy to prevent vascular dysfunction in ischemic organs [17, 18]. However, direct CD47 blockade, such as monoclonal antibody therapy, has been associated with severe hematologic toxicity due to high CD47 expression on erythrocytes [19, 20], highlighting the need for safer, ligand‐targeted approaches that specifically neutralize TSP‐1.

Parallel to these mechanistic insights, advances in autophagy‐based therapeutics have opened new avenues for selective protein degradation. Autophagy‐targeting chimeras (AUTACs) [21, 22], autophagosome‐tethering compounds (ATTECs) [23, 24], and similar platforms demonstrate that controlled activation of the autophagy‐lysosome pathway can efficiently eliminate pathological proteins. Nanoparticles have also been shown to modulate autophagy through their physicochemical properties [25]. Among them, poly (lactic‐co‐glycolic acid) (PLGA) nanoparticles stand out for their excellent biocompatibility and intrinsic ability to induce autophagy, which has been exploited to promote protein degradation in cancer therapy [26].

In this study, we designed TAX2‐functionalized PLGA nanoparticles (TAX2‐NPs) composed of PLGA and DSPE‐PEG‐Mal. TAX2 is a cyclic peptide derived from the CD47 sequence that binds specifically to TSP‐1, competitively blocking its interaction with CD47 [27]. By conjugating TAX2 onto PLGA nanoparticles, we created a biocompatible system capable of capturing locally produced TSP‐1 in ischemic liver tissue and delivering it into macrophages for autophagy‐lysosome‐mediated degradation. This strategy enables selective clearance of extracellular TSP‐1, thereby attenuating endothelial apoptosis and inflammation, and ultimately protecting the liver from IRI. In summary, this study introduces a ligand‐targeted nanotherapeutic approach that integrates TSP‐1 sequestration, inter‐organ trafficking, and autophagy‐driven degradation to achieve precise and safe blockade of the TSP‐1/CD47 signaling pathway. By restoring endothelial homeostasis and attenuating hepatic injury, TAX2‐NPs provide a novel framework for designing nanomaterials capable of selective extracellular protein neutralization in ischemic and inflammatory diseases.

## 2 Results

### 2.1 TAX2‐Functionalized Nanoparticles Specifically Bind and Neutralize TSP‐1 to Prevent Endothelial Cell Apoptosis

**FIGURE 1:**
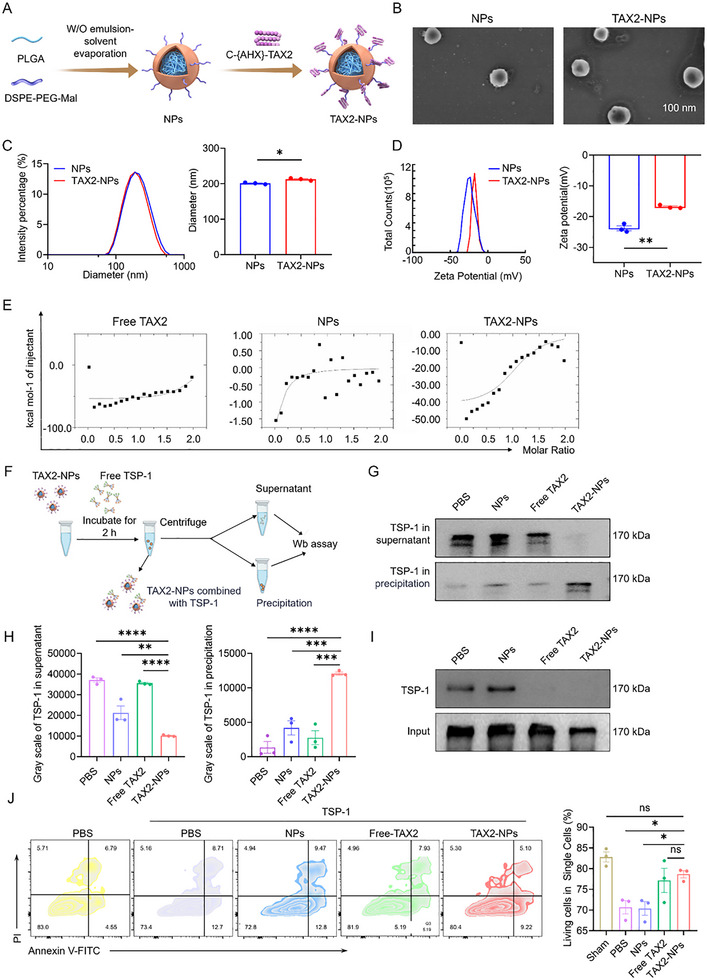
TAX2‐functionalized nanoparticles specifically bind and neutralize TSP‐1 to prevent endothelial cell apoptosis. (A) Schematic diagram of particle synthesis. TAX2 peptide was engineered with a terminal cysteine to enable covalent linkage to DSPE‐PEG‐Mal through a stable thioether bond, yielding TAX2‐functionalized PLGA nanoparticles. (B) Representative scanning electron microscopy (SEM) images of NPs and TAX2‐NPs, scale bar, 100 nm. (C) Average particle size and size distribution of NPs and TAX2‐NPs (*n* = 3 independent preparations) *p < 0.05 by Unpaired Student's *t*‐tests. (D) Zeta potential of NPs and TAX2‐NPs (*n* = 3 independent preparations). **p < 0.01by Unpaired Student's *t*‐tests. (E) Isothermal titration calorimetry (ITC) profiles of TSP‐1 binding to free TAX2 peptide, blank NPs, and TAX2‐NPs. TAX2‐NPs exhibited thermodynamic parameters comparable to free TAX2 (ΔS = −2.04 × 10^3^ cal mol^−1^ deg^−1^ vs. −2.62 × 10^3^ cal mol^−1^ deg^−1^), whereas blank NPs showed negligible interaction (ΔS = 56.9 cal mol^−1^ deg^−1^). Detailed values are summarized in Table S1, Supporting Information. (F) Experimental workflow for Western blot analysis of TSP‐1 binding. Recombinant TSP‐1 was co‐incubated with PBS, free TAX2, NPs, or TAX2‐NPs, followed by centrifugation to separate supernatant and pellet fractions. (G) Representative Western blot images showing TSP‐1 levels in supernatant and pellet fractions. (H) Quantification of TSP‐1 levels in each group (n = 3 independent preparations). (I) Co‐IP was performed using an anti‐CD47 antibody. The bound proteins were eluted, and co‐precipitated TSP‐1 was detected by Western blot. (J) Flow‐cytometric detection of early and late apoptosis in mouse LSECs co‐incubated with TSP‐1 and TAX2‐NPs, free TAX2, NPs, or PBS, and Quantification of survival LSECs (n = 3 independent experiments). *p < 0.05, **p < 0.01, ***p < 0.001, ****p < 0.0001 by one‐way ANOVA followed by Tukey's post hoc test.

Biocompatible PLGA‐based nanoparticles were prepared using a single‐emulsion method by co‐assembling PLGA with DSPE‐PEG‐Mal, which provided surface maleimide (Mal) groups for peptide conjugation. The TAX2 cyclic peptide, derived from the CD47 domain that interacts with TSP‐1, was designed to competitively block the TSP‐1/CD47 interaction with high specificity. By mimicking the native TSP‐1‐binding site of CD47, TAX2 enables selective engagement of TSP‐1 while avoiding the off‐target effects of global CD47 inhibition. TAX2 peptides were covalently linked to the nanoparticle surface via stable thioether bonds formed between cysteine residues on TAX2 and the maleimide groups on DSPE‐PEG‐Mal, yielding TAX2‐functionalized nanoparticles (TAX2‐NPs) capable of specifically binding and sequestering extracellular TSP‐1 (Figure 1A).

Morphological characterization confirmed that TAX2‐NPs maintained a uniform spherical shape with particle size and compared to unmodified nanoparticles, the particle size of TAX2‐NPs was slightly increased, and the zeta potential was elevated. Furthermore, upon binding to free TSP‐1, the particle size further increased and the zeta potential showed a slight rise (Figure 1B–D, Figure S1A,B). Transmission electron microscopy (TEM) imaging confirmed that these changes in particle size and zeta potential were not attributed to particle aggregation (Figure S1C). Quantitative analysis of unbound peptides revealed a coupling ratio of approximately 1:1 between Mal groups and TAX2 molecules, confirming efficient surface conjugation. Isothermal titration calorimetry (ITC) was then performed to evaluate the thermodynamic profiles of TSP‐1 binding to free TAX2, blank NPs, and TAX2‐NPs (Figure 1E, Table S1). Both free TAX2 and TAX2‐NPs exhibited similar exothermic binding curves toward TSP‐1, whereas blank NPs showed negligible interaction. The molar binding entropy of TAX2‐NPs (ΔS = −122 cal^−1^mol^−1 ^deg) was slightly higher than that of free TAX2 (ΔS = −160 cal^−1^mol^−1 ^deg), but several orders of magnitude smaller than that of blank NPs (ΔS = −7.4 × 10^5^ cal^−1^mol^−1 ^deg), indicating that TSP‐1 adsorption on TAX2‐NPs was primarily driven by specific TAX2‐TSP‐1 interactions rather than nonspecific surface effects.

Western blot analysis further verified the specific binding capacity of TAX2‐NPs to free TSP‐1 (Figure 1F–H). Recombinant TSP‐1 was co‐incubated with PBS, free TAX2, blank NPs, or TAX2‐NPs, followed by centrifugation to separate nanoparticle‐bound (pellet) and unbound (supernatant) fractions. Almost no TSP‐1 band was detected in the supernatant of the TAX2‐NPs group, whereas a strong band appeared in the pellet fraction (Figure 1G). Densitometric quantification showed that the TSP‐1 level in the supernatant of the TAX2‐NPs group was approximately one‐twentieth that of the PBS control, while its level in the pellet was nearly 20‐fold higher. In contrast, blank NPs showed only minimal nonspecific adsorption. Meanwhile, co‐immunoprecipitation (CO‐IP) assays confirmed that treatment of mouse LSECs with free TAX2 or TAX2‐NPs abrogated the binding between surface CD47 and TSP‐1 (Figure 1I, Figure S2). These results demonstrate the high binding efficiency and stability of TAX2‐NPs toward free TSP‐1.

To determine whether TAX2‐NPs could block the pro‐apoptotic effect of TSP‐1, mouse LSECs, the primary TSP‐1 targets during liver IRI, were used as a functional model. Under hypoxic conditions, TSP‐1 binding to CD47 inhibits VEGFR2‐VEGF interaction and suppresses AKT‐PI3K signaling, leading to endothelial cell apoptosis [28, 29, 30]. Flow‐cytometric analysis using Annexin V/PI staining revealed that TSP‐1 treatment markedly increased apoptosis and reduced cell viability in LSECs in both the PBS + TSP‐1 and NPs + TSP‐1 groups compared with normal controls. In contrast, the presence of free TAX2 or TAX2‐NPs significantly reduced apoptotic cell populations and restored cell viability (Figure 1J, Figure S3), indicating that TAX2‐NPs effectively neutralize the pro‐apoptotic activity of TSP‐1 by preventing its interaction with CD47.

Collectively, these findings demonstrate that TAX2‐functionalized nanoparticles effectively capture and neutralize extracellular TSP‐1 through specific biomimetic interactions, thereby interrupting the pathological TSP‐1/CD47 signaling and preventing endothelial apoptosis. This biomaterial strategy, which exploits the CD47‐derived TSP‐1‐binding motif of TAX2, provides a precise and biocompatible approach to selectively modulate the TSP‐1/CD47 axis without the off‐target effects associated with global CD47 blockade.

### 2.2 TAX2‐NPs Mediate Inter‐Organ Redistribution of TSP‐1 by Reducing its Hepatic Accumulation and Facilitating its Transport to the Spleen

**FIGURE 2:**
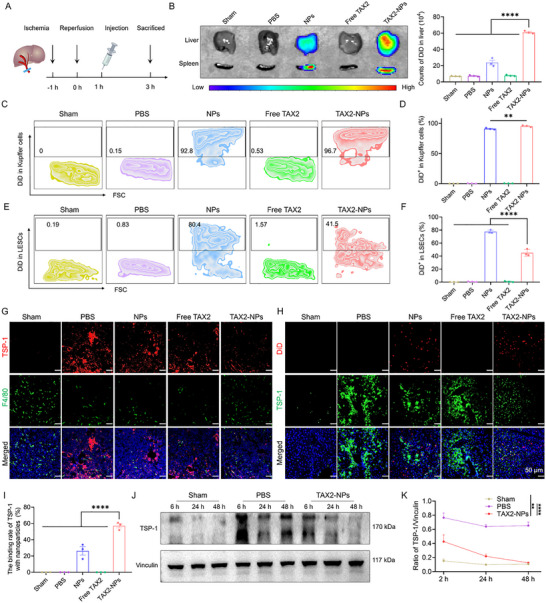
TAX2‐NPs mediate inter‐organ redistribution of TSP‐1 by reducing its hepatic accumulation and facilitating its transport to the spleen. (A) Schematic diagram of the treatment and assay of TSP‐1 in the early stage of murine liver IR. (B) In vivo imaging (IVIS) of nanoparticle accumulation in the murine liver and spleen 2 h after intravenous injection and Quantification of DiD fluorescent dye in the liver (*n* = 3 independent preparations). (C) Flow cytometry detection of the proportion of Kupffer cells in the liver that phagocytosed nanoparticles after tail vein injection of the drug. (D) Quantification of Kupffer cells that phagocytosed particles in mice (n = 3 independent preparations). (E) Flow cytometry detection of the proportion of LSECs in mice that phagocytosed nanoparticles after tail vein injection of the drug. (F) Quantification of LSECs that phagocytosed particles in murine liver (*n* = 3 independent preparations). (G) Confocal microscopy detection of the co‐localization of TSP‐1 and Kupffer cells in the liver, scale bar, 50 µm, (green: F4/80, red: TSP‐1, blue: DAPI). (H) Confocal microscopy detection of the co‐localization of nanoparticles and TSP‐1 in the liver, scale bar,50 µm, (green: TSP‐1, red: DiD, blue: DAPI). (I) Proportion of TSP‐1 co‐localized with nanoparticles (*n* = 3 independent preparations). (J) Representative Western blot images of TSP‐1 levels at different time points in the IRI liver of mice in the sham operation group, PBS or TAX2‐NPs treated group. (K) Quantification of TSP‐1 levels in each group (*n* = 3 independent preparations, normalized to Vinculin, ****p < 0.0001 by Two‐way ANOVA followed by Tukey's post hoc test). *p < 0.05, **p < 0.01, ***p < 0.001, ****p < 0.0001 by one‐way ANOVA followed by Tukey's post hoc test.

To investigate whether TAX2‐NPs could promote systemic clearance and redistribution of TSP‐1 in vivo, a 70% hepatic IRI mouse model was established, in which TSP‐1 expression rapidly increases within 3 h of reperfusion. TAX2‐NPs were intravenously administered at the onset of reperfusion to capture nascent TSP‐1 and prevent its binding to CD47 (Figure 2A). In vivo fluorescence imaging using DiD‐labeled nanoparticles demonstrated that TAX2‐NPs primarily accumulated in the liver and spleen within 2 h post‐injection (Figure 2B, Figure S4). The accumulation of TAX2‐NPs in the IRI liver was significantly increased than that of NPs, whereas no such difference was observed in normal mice. Notably, the overall hepatic accumulation of TSP‐1 pretreated TAX2‐NPs remained comparable to that of unmodified NPs (Figures S5,S6), suggesting that the enhanced retention in IRI livers may be attributed to their interaction with elevated TSP‐1 rather than nonspecific biodistribution differences. Flow‐cytometric analysis further showed that the nanoparticles were mainly internalized by macrophages and endothelial cells (Figure 2C–F), consistent with their roles in TSP‐1 uptake and clearance.

Immunofluorescence staining of IRI liver sections revealed that in the injured liver lobes, TAX2‐NPs reduced the production of TSP‐1 by endothelial cells in the early stage of Liver IRI (Figure S7), and TAX2‐NPs had extensive co‐localization with TSP‐1, with TSP‐1 predominantly localized in macrophages (Figure 2G–I). In contrast, TSP‐1 remained diffusely distributed in the PBS‐, NPs‐, and free TAX2‐treated livers. Western blot analysis confirmed that TAX2‐NPs treatment markedly reduced hepatic TSP‐1 accumulation during the early reperfusion stage (Figure 2J,K). These findings indicate that TAX2‐NPs can effectively capture and reduce extracellular TSP‐1 at the injury site and accelerate its reduction.

Interestingly, substantial accumulation of TAX2‐NPs was also observed in the spleen during the same period. Quantitative analysis showed that the splenic TSP‐1 level in TAX2‐NPs‐treated mice was significantly elevated compared with that of the control groups at 2 h post‐injection (Figure S8). Immunofluorescence analysis revealed strong colocalization of TSP‐1 with splenic macrophages and endothelial cells (Figures S9,S10), moreover, TSP‐1 in the spleen also co‐localizes with TAX2‐NPs (Figure S11). indicating that the captured TSP‐1 was transported from the liver to the spleen via circulation. These observations suggest that the spleen functions as a secondary site for TSP‐1 sequestration and metabolic processing, potentially contributing to its systemic clearance, Confocal imaging results of other major organs showed that there was no increase in TSP‐1 expression in the heart, lung, or kidney (Figure S12).

Collectively, these results uncover a previously unrecognized nanoparticle‐mediated inter‐organ trafficking pathway, in which TAX2‐NPs redirect extracellular TSP‐1 from the injured liver to splenic macrophages and endothelial cells for subsequent degradation. This mechanism highlights the dual function of TAX2‐NPs, as both molecular decoys and systemic shuttles, establishing a novel paradigm for nanoparticle‐enabled redistribution and clearance of pathological extracellular matrix proteins.

### 2.3 TAX2‐NPs Promote Autophagy‐Lysosome‐Mediated Degradation of TSP‐1

**FIGURE 3:**
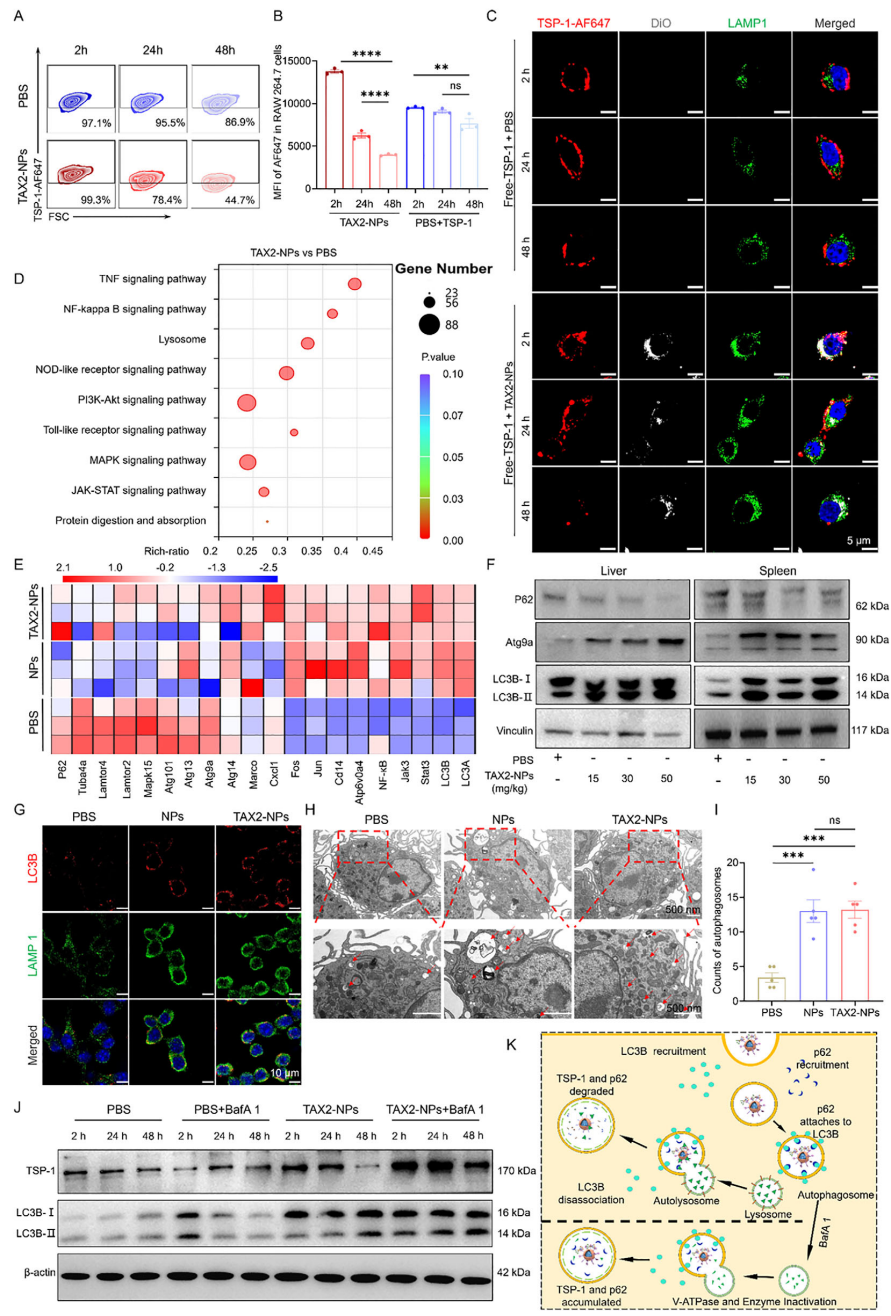
TAX2‐NPs promote autophagy‐lysosome‐mediated degradation of TSP‐1. (A) Flow cytometry was used to detect the TSP‐1 signal in RAW264.7 cells after phagocytosis of TSP‐1 in each group. (B) Quantitative analysis of the average fluorescence intensity of intracellular TSP‐1 in the TAX2‐NPs treatment group (n = 3 independent preparations). (C) Confocal microscopy was used to detect the co‐localization of TSP‐1 and LAMP1, representing late lysosomes, in RAW264.7 cells co‐incubated with TAX2‐NPs‐TSP‐1 for 2‐, 24‐, and 48‐ hours, scale bar, 5 µm (green: LAMP1, red: AF647, white: DiO, blue: DAPI). (D) KEGG enrichment analysis of significantly upregulated autophagy‐related pathways in mouse liver treated with TAX2‐NPs compared with PBS (*p* < 0.05), statistical significance was determined by hypergeometric test and p‐values were corrected for multiple testing (*n* = 3 independent preparations). (E) Heatmap of autophagy‐related genes in liver tissues of normal mice treated with PBS, NPs or TAX2‐NPs for 24 h detected by RNA‐seq (*n* = 3 independent preparations). (F) Representative Western blot images of several autophagy‐related genes (P62, Atg9a, LC3B) in the liver of normal mice 24 h after intravenous injection of different doses of TAX2‐NPs. (G) Confocal microscopy was used to detect the co‐localization of late lysosomes and autophagosomes in RAW264.7 cells treated with PBS/NPs/TAX2‐NPs, scale bar, 10 µm (green: LAMP1, red: LC3B, blue: DAPI). (H) Representative TEM images of mouse bone marrow‐derived macrophages (BMDM) treated with PBS, blank nanoparticles, and TAX2‐NPs for 24 h, with red arrows indicating autophagosomes, scale bar, 10 µm. (I) Quantitative results of the number of autophagosomes in mouse macrophages treated under different conditions (*n* = 5 independent preparations). (J) Representative Western blot images of TSP‐1 and LC3B levels in RAW264.7 cells at different time points after specified treatments. (K) Schematic diagram of the degradation of TSP‐1 after binding with TAX2‐NPs through the autophagy pathway. All Western blot analysis experiments were performed in three independent groups. *p < 0.05, **p < 0.01, ***p < 0.001, ****p < 0.0001 by one‐way ANOVA followed by Tukey's post hoc test.

To elucidate the intracellular fate of TAX2‐NPs after binding to extracellular TSP‐1, recombinant TSP‐1 was labeled with AF647 and co‐incubated with TAX2‐NPs in RAW264.7 macrophages. After two hours of incubation, the medium was replaced with serum‐free medium, and intracellular fluorescence was monitored by flow cytometry at 2‐, 24‐, and 48‐hour post‐treatment (Figure 3A,B). Cells treated with TAX2‐NPs exhibited markedly enhanced AF647 fluorescence compared with controls, reflecting efficient internalization of TSP‐1. The fluorescence intensity gradually decreased over time, indicating that TAX2‐NPs not only facilitated the internalization of TSP‐1 but also mediated its subsequent intracellular degradation. Confocal imaging further revealed pronounced colocalization of internalized TSP‐1 with late endosomal/lysosomal marker (Figure 3C), suggesting that TAX2‐NPs‐bound TSP‐1 is trafficked into the endo‐lysosomal compartment for degradation.

The lysosomal colocalization of TSP‐1 observed in vitro prompted us to examine whether TAX2‐NPs could activate autophagic degradation pathways in vivo. Because lysosomal activity in macrophages is tightly coupled with autophagy signaling to maintain proteostatic homeostasis, we next performed transcriptomic profiling of normal C57BL/6J mice intravenously injected with PBS, NPs or TAX2‐NPs (Figure S13). RNA sequencing revealed robust upregulation of autophagy‐related genes in the livers of TAX2‐NPs‐treated mice compared with controls (Figure 3E). KEGG and GO enrichment analyses confirmed significant activation of multiple autophagy‐associated signaling pathways (Figure 3D, Figure S14). Meanwhile, RNA‐seq analysis showed administration of free TAX2 alone did not alter the expression of autophagy‐related genes. In addition, the presence of free TSP‐1 did not impair the ability of TAX2‐NPs to induce autophagy in cells (Figures S15,S16). Western Blot analysis further validated activation of autophagy at the protein level (Figure 3F). TAX2‐NPs treatment led to a dose‐dependent increase in Atg9a expression and a concomitant reduction in P62 levels, indicating enhanced autophagic flux. The conversion of LC3B‐I to LC3B‐II was also elevated compared with controls, consistent with autophagosome formation, although LC3B‐II levels did not show a strict dose‐response relationship. These findings confirm that TAX2‐NPs activate canonical autophagy signaling and enhance autophagic flux in vivo. Similar transcriptional trends were also observed in splenic tissues (Figure 3F), suggesting that TAX2‐NPs elicit a systemic autophagic response that may facilitate the degradation of captured TSP‐1 across organs. TEM analysis of BMDMs treated with PBS, NPs, or TAX2‐NPs for 24 h revealed numerous double‐membrane vesicles in both NP‐ and TAX2‐NPs‐treated cells (Figure 3H). Quantitative analysis showed a significant increase in the number and diameter of autophagosomes compared with controls (Figure 3I, Figure S17), confirming that nanoparticles themselves can stimulate autophagosome formation, and that TAX2 modification has no impact on this effect.

Mechanistically, autophagy proceeds through LC3B and P62‐dependent pathways, during which cytosolic LC3B‐I is lipidated to form LC3B‐II on the autophagosomal membrane. P62 acts as an adaptor protein linking LC3B to ubiquitinated substrates, which are subsequently sequestered into autophagosomes. Upon fusion with late endosomes, these structures form autolysosomes where cargo proteins are degraded by lysosomal enzymes [31, 32]. Inhibition of lysosomal acidification with bafilomycin A1 prevents this degradation (Figure 3K). To verify that TAX2‐NPs mediate TSP‐1 degradation through this autophagy‐lysosome pathway, RAW264.7 cells were co‐incubated with TSP‐1 and either PBS, NPs, or TAX2‐NPs, followed by immunofluorescence staining for LC3B and late endosome markers. TAX2‐NPs‐treated cells displayed a pronounced increase in LC3B‐positive puncta and strong colocalization between autophagosomes and late endosomes, indicative of active autolysosome formation (Figure 3G). TAX2‐NPs treated RAW cells infected with EGFP‐mcherry‐LC3B virus produced more autophagosomes, and over time, the intensity of EGFP in TAX2‐NPs treated RAW 264.7 cells decreased significantly due to the formation of autolysosomes, indicating smooth flow from autophagosomes to autolysosomes. (Figure S18). Western Blot analysis further demonstrated a time‐dependent accumulation of LC3B‐II accompanied by progressive degradation of TSP‐1, whereas inhibition of lysosomal function with bafilomycin A1 significantly attenuated TSP‐1 degradation (Figure 3J).

Collectively, these findings demonstrate that TAX2‐NPs induce a robust autophagy‐lysosome response that mediates intracellular degradation of the captured TSP‐1. This mechanism highlights a dual‐phase clearance strategy in which TAX2‐NPs first sequester extracellular TSP‐1 via biomimetic binding and subsequently direct it toward autophagy‐dependent degradation within macrophages. The discovery of this nanoparticle‐mediated autophagic clearance pathway provides a mechanistic foundation for targeted elimination of pathological extracellular proteins through engineered nanocarriers.

### 2.4 TAX2‐NPs Preserve the Structural and Functional Integrity of Liver Sinusoidal Endothelial Cells Following IRI

**FIGURE 4:**
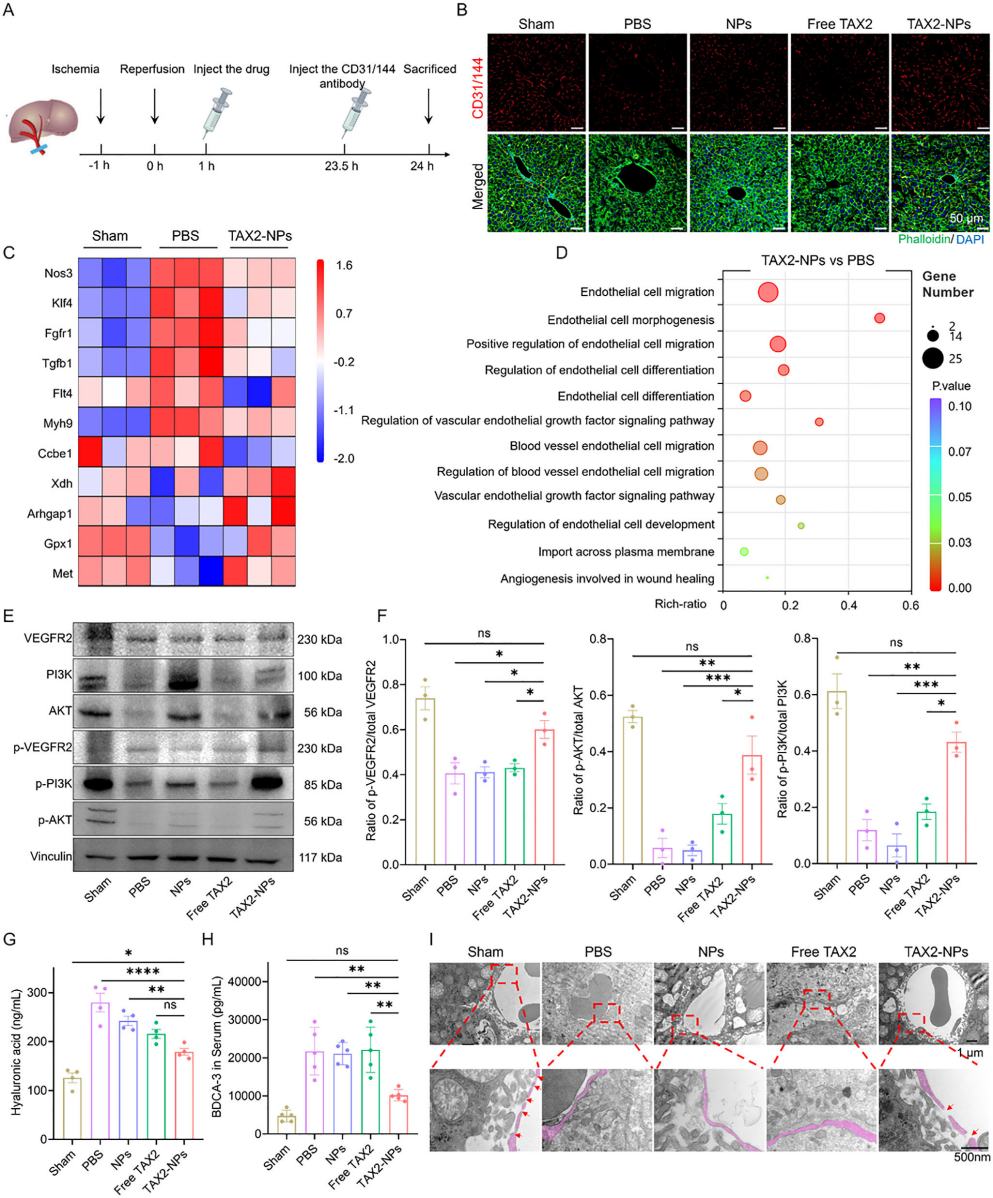
TAX2‐NPs preserve the structural and functional integrity of liver sinusoidal endothelial cells following IRI. (A) Schematic diagram of endothelial cell injury in the mouse liver caused by IR. (B) Confocal microscopy detection of liver endothelial cell survival, scale bar, 50 µm. (Green: Phalloidin, Red: CD31/144, Blue: DAPI). (C) Heatmap of endothelial cell‐related genes in IRI liver tissues from the sham operation group, PBS or TAX2‐NPs treated 24 h later detected by RNA‐seq (*n* = 3 independent preparations). (D) GO enrichment analysis of significantly upregulated endothelial cell‐related pathways (*p* < 0.05) in the murine livers treated with TAX2‐NPs compared with PBS was performed, and statistical significance was determined by hypergeometric test. Meanwhile, the p‐values were corrected for multiple testing (*n* = 3 independent preparations). (E) Representative Western blot images of VEGFR2, PI3K, AKT, and their phosphorylation levels in the IRI liver of sham operation group, PBS or TAX2‐NPs treated mice. (F) Quantitative analysis of phosphorylation levels of VEGFR2, PI3K, and AKT in each group (*n* = 3 independent preparations, normalized to Vinculin). (G) ELISA detection of hyaluronic acid content in serum (*n* = 4 independent preparations). (H) ELISA detection of BDCA‐3 content in serum (*n* = 5 independent preparations). (I) Representative TEM images of liver sinusoids in the IRI liver of mice treated with designated conditions. Purple pseudo‐color indicates liver sinusoidal endothelial cells. Arrows indicate endothelial fenestrae. scale bar, 500 nm. *p < 0.05, **p < 0.01, ***p < 0.001, ****p < 0.0001 by one‐way ANOVA followed by Tukey's post hoc test.

Endothelial cells, particularly LSECs, are among the earliest and most susceptible targets of liver IRI, and their dysfunction serves as a key driver of microvascular collapse and hepatic damage. Protecting endothelial cell integrity is therefore essential for mitigating IRI‐induced hepatic injury. In our study, the results of confocal microscopy indicated that 24 h after injection, TAX2‐NPs could be enriched in the endothelial cells of the IRI liver in mice (Figure 4A, Figure S19). Immunofluorescence staining for CD31 and CD144 confirmed that endothelial continuity and junctional organization were largely preserved in the TAX2‐NPs group (Figure 4B, Figure S20). Transcriptomic profiling further demonstrated that TAX2‐NPs normalized the expression of endothelial function‐related genes, restoring them toward Sham‐operation levels (Figure 4C,D).

Under physiological conditions, VEGFR2 (vascular endothelial growth factor receptor 2) serves as a central regulator of endothelial survival and vascular homeostasis through activation of the PI3K‐AKT signaling cascade, which maintains cytoskeletal stability, nitric oxide production, and anti‐apoptotic signaling. However, prior studies have shown that TSP‐1/CD47 engagement directly inhibits VEGFR2 phosphorylation and downstream AKT activation [28], thereby amplifying oxidative stress and promoting endothelial cell apoptosis [33]. Given this established mechanism, we investigated whether TAX2‐NPs could protect VEGFR2 signaling suppressed by TSP‐1/CD47 interaction during hepatic IRI. Western blot analysis revealed that TAX2‐NPs treatment significantly restored the activation of the VEGFR2‐PI3K‐AKT signaling axis in the liver following ischemia‐reperfusion injury. (Figure 4E,F). Although the total protein levels of VEGFR2, PI3K, and AKT in the TAX2‐NPs group were slightly reduced compared with the Sham group, their phosphorylated forms (p‐VEGFR2, p‐PI3K, and p‐AKT) remained largely preserved, resulting in a sustained phosphorylation ratio indicative of intact signaling activity. In contrast, PBS‐, NPs‐, and free TAX2‐treated livers showed a marked decrease in the phosphorylation of VEGFR2, PI3K, and AKT, indicating inactivation of this pro‐survival pathway despite comparable total protein abundance (Figure 4E). Quantitative analysis confirmed that TAX2‐NPs effectively maintained the phosphorylation ratios (p/t‐VEGFR2, p/t‐PI3K, and p/t‐AKT (Figure 4F). These results suggest that by neutralizing extracellular TSP‐1, TAX2‐NPs effectively preserve VEGFR2‐PI3K‐AKT signaling axis activity by preventing TSP‐1/CD47‐mediated dephosphorylation, thereby maintaining endothelial cell viability under oxidative stress.

To further evaluate the functional protection of LSECs, we quantified markers of endothelial injury and metabolic competence. TAX2‐NPs‐treated mice exhibited significantly lower plasma hyaluronic acid (a surrogate for LSEC functional impairment [34]; Figure 4G) and reduced circulating BDCA‐3 (a marker of endothelial cell damage [35]; Figure 4H). Transmission electron microscopy (TEM) of hepatic tissue 24 h after reperfusion showed that LSECs in the TAX2‐NPs‐treated mice retained abundant and well‐organized fenestrae, preserving the characteristic sieve plate morphology and sinusoidal permeability (Figure 4I). In contrast, control groups displayed extensive defenestration and basement membrane formation, consistent with LSEC capillarization and endothelial dedifferentiation under oxidative stress [36]. Together, these findings indicate that TAX2‐NPs not only protect endothelial structure but also maintain endothelial function, in part by restoring VEGFR2‐AKT signaling disrupted by TSP‐1/CD47 interaction, thereby maintaining both structural and functional homeostasis of LSECs after liver IRI.

Finally, in TSP‐1‐ mice, TAX2‐NPs failed to provide additional protection beyond that of NPs, and endothelial injury remained minimal in all groups (Figure S21). These results confirm that the therapeutic effect of TAX2‐NPs depends specifically on TSP‐1 neutralization and that inhibition of the TSP‐1/CD47‐VEGFR2 axis is central to their endothelial protective mechanism.

### 2.5 TAX2‐NPs Preserve Hepatic Apoptosis and Tissue Injury During IR by Suppressing Inflammation and Oxidative Stress

**FIGURE 5:**
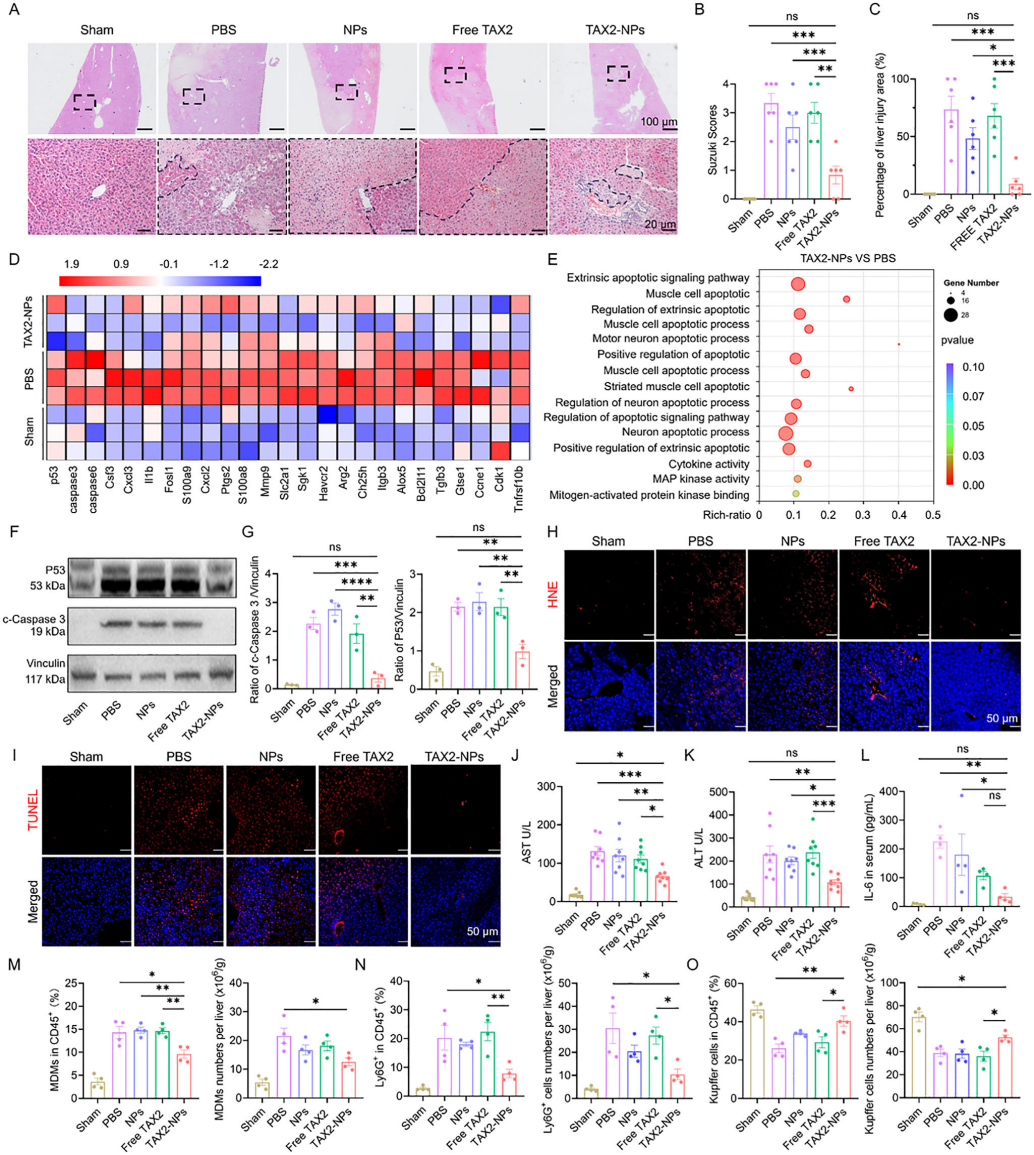
TAX2‐NPs preserve hepatic apoptosis and tissue injury during IR by suppressing inflammation and oxidative stress. (A) Representative images of H&E staining of IRI liver in mice, scale bar, 20 µm. (B) Suzuki score of H&E staining of IRI liver in mice (*n* = 6 independent preparations). (C) Quantification of injury area in H&E staining of IRI liver in mice (*n* = 6 independent preparations). (D) GO enrichment analysis of significantly downregulated apoptosis‐related pathways in the IRI liver of mice treated with TAX2‐NPs compared with PBS (*p* < 0.05), statistical significance was determined by hypergeometric test and p‐values were corrected for multiple comparisons (n = 3 independent preparations). (E) Heatmap of apoptosis‐related genes in IRI liver tissues 24 h after reperfusion in sham operation group, PBS or TAX2‐NPs treated mice detected by RNA‐seq (*n* = 3 independent preparations). (F) Representative Western blot images of P53 and c‐Caspase 3 levels in the IRI liver 24 h after reperfusion in the designated treatment groups. (G) Quantitative analysis of P53 and c‐Caspase 3 levels (n = 3 independent preparations, normalized to Vinculin). (H) Representative images of HNE staining of the liver 24 h after reperfusion injury in each group captured by confocal microscopy, scale bar, 50 µm, (red: HNE, blue: DAPI). (I) Representative images of TUNEL staining of the liver 24 h after reperfusion in each group captured by confocal microscopy, scale bar, 50 µm, (red: TUNEL, blue: DAPI). (J) Quantification of AST in mouse serum by ELISA (*n* = 8 independent preparations). (K) Quantification of ALT in mouse serum by ELISA (*n* = 8 independent preparations). (L) Quantification of IL‐6 in mouse serum by ELISA (*n* = 4 independent preparations). (M) Flow cytometry detection of the proportion of MDMs infiltrating the liver and the number of MDMs per unit mass of liver 24 h after reperfusion (*n* = 3 independent preparations). (N) Flow cytometry detection of the proportion of neutrophils infiltrating the liver and the number of neutrophils per unit mass of liver 24 h after reperfusion (n = 3 independent preparations) (O) Flow cytometry was used to detect the proportion of surviving Kupffer cells and the number of surviving Kupffer cells in the liver 24 h after reperfusion (n = 4 independent preparations). *p < 0.05, **p < 0.01, ***p < 0.001, ****p < 0.0001 by one‐way ANOVA followed by Tukey's post hoc test.

To evaluate the therapeutic efficacy of TAX2‐NPs against hepatic IRI, a murine partial hepatic IRI model was established, and nanoparticles were administered one hour after reperfusion (Figure S22). Histological and molecular analyses were performed 24 h later. H&E staining revealed severe lobular disorganization, inflammatory infiltration, and hepatocellular ballooning in the IRI control group, whereas TAX2‐NPs treatment markedly preserved hepatic architecture, with substantially reduced necrotic regions (Figure 5A, Figure S23). Quantitative assessment showed significant reductions in both Suzuki scores and total injury area compared with PBS‐, NPs‐, and free TAX2‐treated controls (Figure 5B,C), indicating robust attenuation of tissue damage. Transcriptomic profiling of ischemic livers confirmed widespread suppression of apoptosis‐related gene networks in TAX2‐NPs‐treated mice (Figure 5D, Figure S24A, B). KEGG and GO enrichment analyses further revealed significant downregulation of pathways associated with apoptosis and inflammation (Figure 5E, Figure S24C), consistent with Western blot findings showing reduced expression of cleaved Caspase‐3 and P53 (Figure 5F,G). Because endothelial injury and oxidative stress are major amplifiers of IRI, hepatic oxidative damage was next examined. 4‐HNE immunostaining revealed extensive lipid peroxidation in untreated IRI livers, which was markedly reduced following TAX2‐NPs treatment (Figure 5H, Figure S25), suggesting preservation of endothelial eNOS activity and redox homeostasis. Consistently, TdT‐mediated dUTP Nick‐End Labeling (TUNEL) staining demonstrated a sharp decrease in apoptotic hepatocytes in TAX2‐NPs‐treated mice compared with controls (Figure 5I, Figure S26), confirming the broad cytoprotective effects of this intervention.

At the systemic level, TAX2‐NPs‐treated mice exhibited serum ALT and AST levels comparable to those of sham‐operated animals (Figure 5J,K), indicating effective protection against hepatocellular necrosis. Circulating IL‐6, a key proinflammatory cytokine predominantly secreted by activated Kupffer cells, infiltrating monocyte‐derived macrophages, and under stress conditions liver sinusoidal endothelial cells [37, 38], was also markedly reduced in TAX2‐NPs‐treated mice (Figure 5L). The concurrent suppression of IL‐6 and tissue damage suggests that TAX2‐NPs effectively mitigate the hepatic inflammatory milieu and cytokine activation. Flow cytometric profiling of hepatic immune populations revealed a pronounced reduction in CD11b^+^F4/80^med^ monocyte‐derived macrophages (MDMs) infiltration and reduced the infiltration of Ly6G^+^ neutrophils. following TAX2‐NPs treatment (Figure 5M,N, Figure S27). In contrast, the proportion of resident Kupffer cells (CD11b^+^F4/80^hi^) was largely preserved; the apoptosis induced by IRI of tissue‐resident Kupffer cells [39] is reduced. (Figure 5O, Figure S27). These data indicate that TAX2‐NPs suppress the recruitment and activation of peripheral inflammatory cells while maintaining the viability and function of tissue‐resident macrophages, thereby interrupting the feed‐forward inflammatory cascade characteristic of IRI.

Finally, to assess the systemic safety of TAX2‐NPs, histological examinations of major organs, including the heart, lungs, spleen, and kidneys, were performed using H&E staining 24 h post‐treatment (Figure S28). No observation of tissue necrosis, inflammatory infiltration, vascular congestion, or structural abnormalities was observed in TAX2‐NPs‐treated mice compared with PBS or sham controls. The architecture of cardiomyocytes, alveolar septa, splenic white and red pulp, and renal glomeruli and tubules remained intact, confirming the absence of off‐target toxicity. Collectively, these findings demonstrate that TAX2‐NPs achieve potent hepatoprotection through simultaneous suppression of apoptosis, oxidative stress, and inflammation, while exhibiting excellent systemic biocompatibility.

## 3 Discussion

IRI is a major complication in liver transplantation and surgery, where early microvascular injury and inflammation lead to irreversible tissue damage. The TSP‐1/CD47 axis plays a central role in this process. Although blockade of this pathway shows therapeutic promise [40, 41], most current approaches directly target CD47 with antibodies or peptides, which cause severe off‐target effects such as anemia and hemagglutination due to CD47 expression on red blood cells. In this study, we introduce a biomimetic nanotherapeutic platform, TAX2‐functionalized PLGA nanoparticles, that selectively captures and neutralizes extracellular TSP‐1, thereby indirectly inhibiting the TSP‐1/CD47 interaction. TAX2, a cyclic peptide derived from the CD47 extracellular domain sequence (SQLLKGD), mimics the native CD47 binding interface and competitively binds TSP‐1 with high affinity [42]. Conjugation of TAX2 onto biocompatible nanoparticles composed of PLGA and DSPE‐PEG‐Mal yielded a stable, biodegradable system capable of sequestering TSP‐1 in situ. This ligand‐interception strategy differs fundamentally from receptor‐targeted approaches: instead of blocking CD47 globally, it eliminates the pathological ligand locally, achieving targeted inhibition without hematologic toxicity.

Mechanistically, TAX2‐NPs exert multifaceted protection against hepatic IRI by intercepting, redistributing, and degrading TSP‐1. First, TAX2‐NPs effectively capture TSP‐1 produced within the ischemic liver microenvironment, competitively blocking its interaction with CD47 on LSECs. This early interception halts the downstream apoptotic and inflammatory signaling cascade, preserving VEGFR2 phosphorylation and maintaining PI3K‐AKT pathway activity. Second, TAX2‐NPs mediate inter‐organ trafficking of TSP‐1, transferring the captured ligand from the injured liver to the spleen for clearance. This previously unrecognized phenomenon reveals that nanoparticles can function as molecular shuttles, redirecting pathological extracellular proteins to degradative sites in extrahepatic tissues, a clearance mechanism not achievable by soluble peptides or antibodies. Third, TAX2‐NPs activate the autophagy‐lysosome degradation pathway in macrophages, promoting the breakdown of internalized TSP‐1. As shown in Figure 3, TAX2‐NPs treatment upregulated Atg9a and LC3B‐II while reducing P62 levels, indicating enhanced autophagic flux. This nanoparticle‐triggered autophagic response defines a new paradigm of nanomaterial‐mediated selective protein degradation in vivo.

These synergistic mechanisms converge to preserve the structural and functional integrity of hepatic sinusoidal endothelium. By neutralizing TSP‐1, TAX2‐NPs prevent endothelial cell apoptosis and maintain fenestrations and sinusoidal permeability, thereby safeguarding microvascular exchange and preventing secondary hepatocellular injury. Functionally, this protection translates into sustained VEGFR2‐PI3K‐AKT signaling and restored vascular homeostasis. Consistent with this, TSP‐1‐ mice exhibited neither IRI‐induced endothelial damage nor therapeutic benefit from TAX2‐NPs, confirming that the observed protection is specifically dependent on TSP‐1 neutralization rather than nonspecific nanoparticle effects.

At the tissue level, TAX2‐NPs treatment significantly reduced oxidative stress, inflammatory cytokine release, and immune cell infiltration, hallmarks of the amplification loop in hepatic IRI. The marked reduction of IL‐6, neutrophil, and monocyte‐derived macrophage infiltration, together with the preservation of Kupffer cell viability, indicates that TAX2‐NPs disrupt the endothelial‐immune crosstalk that perpetuates ischemic inflammation. Consequently, hepatocellular apoptosis and necrosis were markedly attenuated, as reflected by decreased TUNEL positivity and normalization of serum ALT/AST levels. Beyond their therapeutic efficacy, TAX2‐NPs displayed excellent systemic biosafety. Histological examination of the heart, lungs, spleen, and kidneys revealed no evidence of necrosis, vascular congestion, or inflammatory infiltration, confirming the high biocompatibility of this PLGA‐based nanoplatform. The biodegradable nature and clinical familiarity of PLGA further strengthen the translational potential of TAX2‐NPs for ischemic organ protection.

**FIGURE 6:**
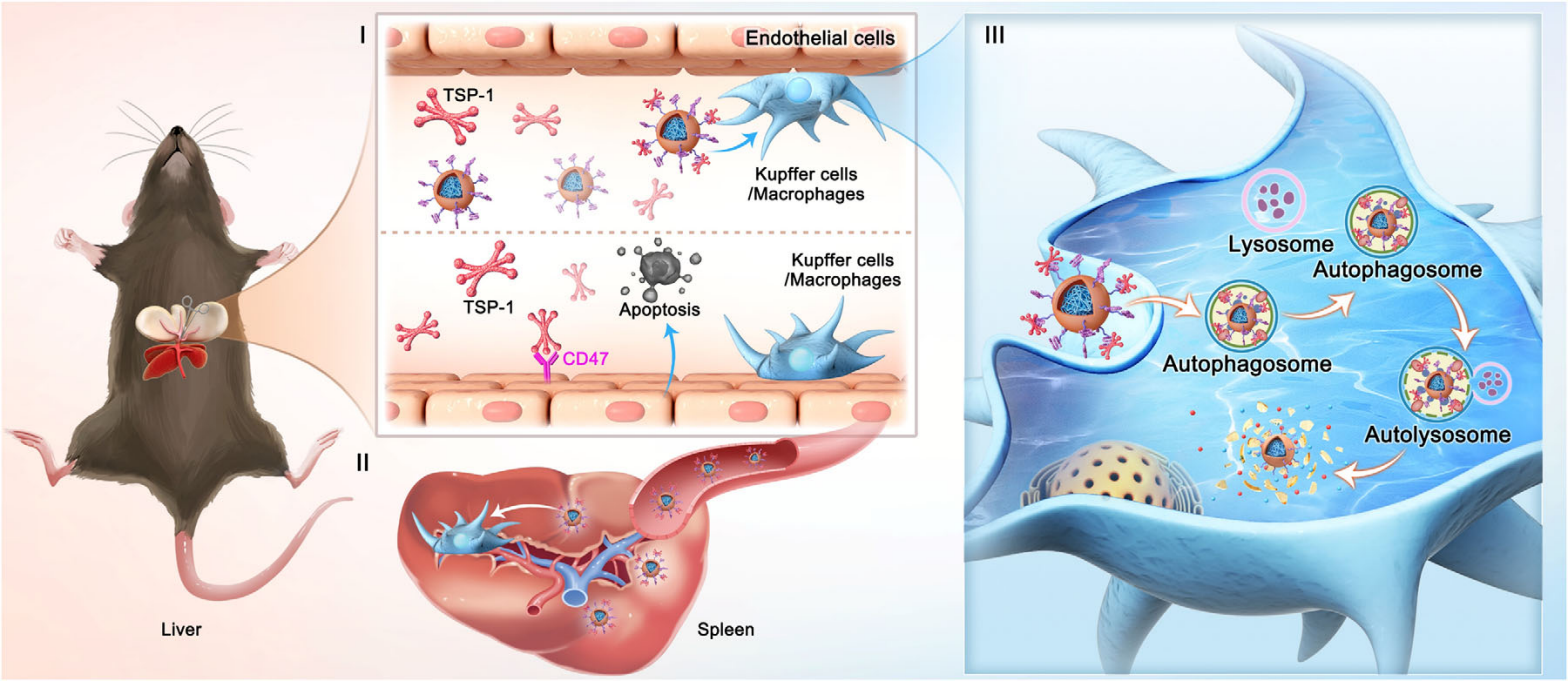
TAX2‐NPs inhibit TSP‐1 binding to CD47, protect liver endothelial cells, and reduce liver injury after ischemia‐reperfusion. (I). TAX2‐NPs competitively bind TSP‐1 with CD47 by mimicking the extracellular binding domain of CD47, thereby blocking the TSP‐1‐CD47 signaling pathway and inhibiting endothelial cell apoptosis. (II)TSP‐1 bound by TAX2‐NPs was transported to the spleen and internalized by splenic macrophages. (III). The TSP‐1 bound by TAX2‐NPs is internalized by macrophages and completely degraded via the autophagy pathway.

In summary, this study establishes a safe and effective nanotherapeutic strategy for targeted degradation of TSP‐1 in ischemic livers. By integrating ligand sequestration, inter‐organ transport, and autophagy‐driven degradation, TAX2‐NPs achieve precise neutralization and clearance of TSP‐1, thereby preserving both endothelial and parenchymal integrity during IRI (Figure 6). This ligand‐interception concept provides a generalizable framework for designing nanotherapeutics that achieve selective extracellular protein clearance. Future optimization, such as surface incorporation of ubiquitin‐mimetic or lysosome‐targeting motifs, may further enhance autophagic degradation efficiency and extend this approach to other TSP‐1/CD47‐associated pathologies, including fibrosis, cardiovascular ischemia, and immune‐mediated disorders.

## 4 Conclusion

In this study, we developed TAX2‐functionalized nanoparticles as a biomimetic nanoplatform for targeted interception and degradation of TSP‐1 in ischemic tissues. TAX2‐NPs specifically capture TSP‐1 locally released from damaged liver microenvironments and deliver it to macrophages for autophagy‐lysosome‐mediated degradation. By eliminating extracellular TSP‐1 and blocking its interaction with CD47, TAX2‐NPs effectively prevent endothelial apoptosis and mitigate ischemia‐reperfusion–induced liver injury. These findings establish a mechanistically grounded and biocompatible nanotherapeutic approach for controlling TSP‐1‐driven vascular injury and highlight a broader strategy for selective extracellular protein clearance in ischemic and inflammatory diseases.

## 5 Experimental Section/Methods

### 5.1 Materials

Poly (D, L‐lactic‐co‐glycolide) (PLGA, MW 7,000‐17,000) was purchased from Sigma‐ Aldrich (St. Louis, MO, USA), DSPE‐PEG‐Mal (MW: 5000) was purchased from Xi'an Ruixi Biological Technology Co., Ltd. (Xi'an, China), TAX2 {CEVSQLLKGDAC‐(AHX)‐C} peptides were purchased from GL Biochem Corporation.Ltd. (Shanghai, China) DAPI‐Fluoromount‐G was purchased from Southern Biotechnology, Associates Inc. (Birmingham, Alabama, USA). Recombinant mouse TSP‐1 protein (carrier‐free) was purchased from R&D Systems (Minneapolis, MN, USA), Annexin V/propidium iodide (PI) staining kit was purchased from eBioscience (SanDiego, CA, USA). ALT/AST determination kit were purchased from Nanjing Jiancheng Bioengineering Institute (Nanjing, China). 1,1'‐dioctadecyl‐3,3,3',3'‐tetramethylindodicarbocyanine, 4‐chlorobenzenesulfonate salt (DiD), Alexa Fluor 647 NHS Ester, Goat anti‐Rabbit IgG (H+L) Secondary Antibody (HRP), and TUNEL Assay kit were purchased from Biyuntian Biotechnology,Co., Ltd. (Shanghai, China). Rabbit anti‐TSP‐1 antibody (ab85762),Rabbit anti‐LC3b antibody (ab192890), Rabbit anti‐sqstm1 antibody (5114T), Rabbit anti‐Atg9a antibody (ab108338), phospho‐VEGF Receptor 2 (Tyr1175) (19A10) Rabbit mAb(2478), Phospho‐PI3 Kinase p85 (Tyr458)/p55 (Tyr199) (E3U1H) Rabbit mAb(17366S), VEGF Receptor 2 (55B11) Rabbit mAb (2479S), Rabbit anti‐PI 3 Kinase catalytic subunit gamma antibody(ab302958), Rabbit anti‐AKT1 (phospho S473) antibody (ab81283), phospho‐Akt (Ser 473) (D9E) XP Rabbit mAb (4060), CD47 antibody (PA5‐116827).

### 5.2 Cell Culture

The Mouse macrophage cell line RAW264.7 was purchased from American Type Culture Collection (ATCC). The LSECs were purchased from Changchun Kelebo Biotechnology Co., Ltd. These cells were maintained in Endothelial cell culture medium or DMEM medium supplemented with 10% Fetal Bovine Serum (FBS), 1% L‐glutamine, and 1% penicillin‐streptomycin, and incubated at 37°C incubator with 5% CO_2_.

### 5.3 Animals

Female C57BL/6J mice (8‐10 weeks) were obtained from Charles River (Beijing, China) and female B6.129S2‐*Thbs1^1Hyn^*/J (TSP‐1‐) from Jackson Laboratory (Bar Harbor, ME, USA) housed in a specific pathogen‐free environment with free access to food and water. All animals’ protocols were reviewed and approved by the Institutional Animal Care and Use Committee of the First Hospital of Jilin University, and all experiments were performed in accordance with the protocols (JDYY20240534).

### 5.4 Liver IRI Model

The mice were fasted for 12 h before the operation but had free access to water. Female C57BL/6J or TSP‐1‐ mice were anesthetized with sodium pentobarbital and maintained at body temperature at 37°C. The portal vein and hepatic artery of the middle and left lobes were clamped with non‐invasive vascular clamps to cause approximately 70% liver ischemia. After 1 h, the vascular clamps were quickly removed, and the liver tissue color became rosy, confirming blood reflow. One hour after reperfusion, PBS, free TAX2, NPs or TAX2‐NPs (1.25 mg kg^−1^ body weight TAX2 peptides per mouse) were injected into the tail vein of the mice with liver IRI. The mice were sacrificed at the designated time points and samples were collected.

### 5.5 Preparation and Characterization of TAX2‐NPs

PLGA NPs were prepared by a W/O emulsion‐solvent evaporation technique using PLGA (16 mg mL^−1^ dissolved in trichloromethane) and DSPE‐PEG‐Mal (10 mg mL^−1^ dissolved in ultrapure water) as previously described [43]. The TAX2 peptides were conjugated to the surface of PLGA NPs through a reaction between the cysteine on peptides and Maleimide groups on PLGA NPs at 4°C overnight. The size and zeta potential of the nanoparticles were determined by dynamic light scattering (Zetasizer Nano ZS90, Malvern Instruments, Southborough, UK) at 25°C, and the morphology was examined by a scanning electron microscope (HITACHI SU800, HITACHI, Tokyo, Japan) at 3 kV.

### 5.6 TAX2‐NPs Bind to TSP‐1 In Vitro

Recombinant mouse TSP‐1 protein was admixed with AF647 fluorescent dye at a molar ratio of 1:1 and agitated at 4°C on a magnetic stirrer for 12 h and subsequently introduced into a 3.5 kDa regenerated cellulose dialysis bag. The TSP‐1‐AF647 was subjected to dialysis in PBS to eliminate the unbound AF647 fluorescence. Subsequently, TSP‐1‐AF647 was combined with TAX2‐NPs at a molar ratio of 1:1 and incubated at 4°C on a shaker for 2 h. The mixture was centrifuged at 30,000 rpm, 4°C, for 30 min, and subsequently re‐suspended in PBS to obtain TSP‐1‐TAX2‐NPs.

### 5.7 Transmission Electron Microscope (TEM)

Cells or tissues were collected at the indicated time points and fixed in glutaraldehyde for 24 h. After fixation, samples were dehydrated, embedded, and sectioned into ultrathin slices using an ultramicrotome. The sections were then mounted on copper grids and imaged using an HT7800 transmission electron microscope.

### 5.8 Isothermal Titration Calorimetry (ITC)

To assess the interaction between free TAX2, TAX2‐NPs, and TSP‐1, ITC experiments were conducted using an ITC200 at 27°C. All titrations were carried out at 27°C in PBS at a stirring speed of 300 rpm. Control experiments were performed by titrating the titrant into the buffer to correct for dilution heat. At 27°C, 400 µL of TAX2‐NPs/Free‐TAX2/PBS (TAX2 peptide concentration of 10 µg mL^−1^) was added to the sample cell, and 20 µL of TSP‐1 at a concentration of 100 µg mL^−1^ was added to the injection cell, with a titration interval of 90 s.

### 5.9 The Pull‐Down Experiment of TAX2‐NPs on TSP‐1

TSP‐1‐AF647 was mixed with free‐TAX2 or TAX2‐NPs (at a molar ratio of 1:10) in 50 µL PBS, or with an equimolar number of nanoparticles or the same volume of PBS (as a control), and incubated at 37°C for 2 h. The mixture was then centrifuged at 20 000 g for 20 min. The pellet were resuspended in 150 µL of PBS and subjected to protein denaturation. Both the unbound TSP‐1‐AF647 in the supernatant and the TSP‐1‐AF647 bound to TAX2‐NPs in the pellet were analyzed by Western blotting, with a loading volume of 10 µL for each sample.

### 5.10 Co‐Immunoprecipitation (Co‐IP)

RAW 264.7 cells were co‐incubated with TSP‐1 in the presence of PBS, NPs, free TAX2, or TAX2‐NPs for 6 h. Culture medium and cell lysates were combined to capture total TSP‐1, and total protein was subsequently extracted. The extracted proteins were incubated with either IgG or CD47 antibodies at 4°C with gentle rotation for 24 h to form immune complexes. Protein A/G magnetic beads were then used to capture the immune complexes, which were eluted and denatured. TSP‐1 associated with CD47 and total TSP‐1 levels were analyzed by Western blotting.

### 5.11 Analysis of the Experiment on TSP‐1‐Induced Apoptosis of Mouse Liver Sinusoidal Endothelial Cells

LSECs were inoculated in 24‐well plates at a density of 5 × 10^4^ cells per well, using the specialized complete medium for endothelial cells, and incubated overnight at 37°C with 5% CO_2_. TSP‐1‐AF647, Free‐TAX2 + TSP‐1‐AF647, NPs + TSP‐1‐AF647, and TSP‐1 adsorbed by TAX2‐NPs (TSP‐1‐TAX2‐NPs), with the final concentration of TSP‐1 being 20 µg mL^−1^. The annexin V/propidium iodide (PI) staining kit was employed, and cell apoptosis was detected by flow cytometry. The data were analyzed via FlowJo software (TreeStar, San Carlos, CA, USA).

### 5.12 Hematoxylin and Eosin Staining and Suzuki Score of the Liver

The tissues were fixed with 4% paraformaldehyde and sectioned to a thickness of 3.5 µm, followed by hematoxylin‐eosin (H&E) staining. The slides were observed and photographed using an optical microscope (IX71, Olympus, Tokyo, Japan). As previously described, the extent of IRI was assessed using a liver lesion scoring system. The scoring was determined based on the percentage of liver injury area formed by factors such as cellular structure disruption, occurrence of fractures or collapses, cellular edema, nuclear pyknosis and cytoplasmic hyperchromasia in some cells, necrosis, and inflammatory cell infiltration in randomly selected non‐overlapping fields of view (200×). The scoring method was as follows: 0, none; 1%, 1%–10%; 2&, 11%–25%; 3%, 26%–45%; 4%, 46%–75%; 5%, 76%–100%.

### 5.13 RNA Sequencing and Data Analysis

24 h after administration to normal mice injected with TAX2‐NPs/PBS or liver IRI mice, the liver or ischemic side liver was collected, total RNA was extracted. RNA concentration was measured using the Qubit RNA Assay Kit on a Qubit 2.0 Fluorometer (Life Technologies, USA). RNA integrity was assessed with the RNA Nano 6000 Assay Kit on the Agilent 5400 system (Agilent Technologies, USA). Strand‐specific mRNA libraries were constructed using the NEBNext Ultra RNA Library Prep Kit for Illumina (NEB, USA, Cat# E7530L), following the manufacturer's recommendations. cDNA fragments of preferentially 300–500 bp in length were selected using the AMPure XP system (Beckman Coulter, USA), and library amplification was performed on an ABI2720 PCR instrument (Applied Biosystems, USA). The qualified libraries were sequenced by Genewiz and Novogene on an Illumina NovaSeq X Plus platform (Illumina, USA) using a 2 × 150 bp paired‐end configuration.

Raw sequencing data (Fastq format) were processed with Cutadapt (v1.9.1) to obtain high‐quality clean reads by removing adapter sequences and low‐quality bases (Phred score cutoff: 20; error rate: 0.1; adapter overlap: 1 bp; minimum length: 75; proportion of N: 0.1). The clean reads were then aligned to the reference genome using HISAT2 (v2.0.1). Differential gene expression analysis was performed using the DESeq2 package in R/Bioconductor, which incorporates data‐driven prior distributions for the estimation of dispersion and log2 fold changes. Genes with an adjusted p‐value (padj) of less than 0.05 were considered statistically significantly differentially expressed.

### 5.14 Immunofluorescence Histology

The liver tissues were fixed in 4% paraformaldehyde solution at 4°C overnight, immersed in 30% sugar solution overnight, and then sectioned (4 µm), followed by counterstaining with DAPI, rabbit anti‐TSP‐1, rabbit anti‐4‐HNE, and goat anti‐rabbit IgG secondary antibody (AF555).

Cell climbing slices were fixed with 4% paraformaldehyde at room temperature for 20 min, and then the cells were permeabilized with 0.1% Triton X‐100, followed by counterstaining with DAPI and LAMP1‐AF555. The staining of endothelial cells in the liver of mice was carried out in vivo staining by tail vein injection of a dose of 0.5 µg g^−1^ of CD31/CD144‐AF647 30 min before the treatment of mice.

For the analysis of cell apoptosis, paraffin sections were dewaxed, subjected to antigen retrieval by heating in citrate buffer at 100°C for 3 min, and then apoptotic cell detected by the TUNEL Assay kit in accordance with the manufacturer's instructions. Images were acquired using a Zeiss LSM 880 confocal laser scanning microscope (Carl Zeiss, Oberkochen, Germany) with a 20× objective, and analyzed using ImageJ.

### 5.15 Western Blot Analysis

Liver tissues were homogenized in a radioimmunoprecipitation buffer containing 1% phenylmethylsulfonyl fluoride using a frozen lysis buffer. After 30 min of ice incubation, the lysates were collected by centrifugation at 13 000 rpm at 4°C for 15 min. The protein concentration was determined using a bicinchoninic acid protein assay kit. After electrophoresis separation on a 10% SDS—polyacrylamide gel, the proteins were transferred onto a polyvinylidene fluoride membrane.TSP‐1 was detected using a rabbit anti‐TSP‐1 antibody, AKT using a rabbit anti‐AKT antibody, cleaved Caspase 3 using a rabbit anti‐ cleaved Caspase 3 antibody, P53 using a rabbit anti‐P53 antibody, β‐actin using a mouse anti‐β‐actin antibody, vinculin using a mouse anti‐vinculin antibody, VEGFR2 using a rat anti‐VEGFR2 antibody, and HRP‐labeled goat anti‐rabbit IgG (H+L) secondary antibody, HRP‐labeled goat anti‐rat IgG (H + L) secondary antibody, and HRP‐labeled goat anti‐mouse IgG (H + L) secondary antibody were employed for visualization with an enhanced chemiluminescence substrate (Thermo Fisher Scientific). The gray values of the Western blot bands were statistically analyzed by ImageJ software.

### 5.16 In Vivo Biodistribution of TAX2‐NPs

Briefly, 2 h after intravenous injection of DiD‐TAX2‐NPs, or other control groups, LSECs and macrophages of C57BL/6J mice were isolated using the Liver Dissociation Kit, in accordance with the manufacturer's instructions, and the cellular uptake of DiD‐TAX2‐NPs in vivo was analyzed by flow cytometry.

For fluorescence microscopic observation, the major organs (heart, lung, liver, spleen, and kidney) of mice were fixed with 4% paraformaldehyde and immersed in 30% sucrose solution. The mouse liver was frozen at the optimal cutting temperature (O.C.T.) and sectioned into 4 µm slices, which were stained with PE anti‐mouse F4/80 monoclonal antibody (Bio Legend) and Alexa Fluor 488 anti‐mouse phalloidin, and counterstained with DAPI. For endothelial cell staining, CD31/CD144 antibody was intravenously injected via the tail vein 30 min before handling the mice. The cellular uptake of DiD‐TAX2‐NPs was observed using LSM880 (Zeiss). For IVIS imaging, C57BL/6J mice were intravenously injected with DiD‐TAX2‐NPs, DiD‐NPs, or PBS control one hour after liver IRI. The major organs (lung, heart, liver, spleen, and kidney) were harvested 2 h after the administration of DiD‐TAX2‐NPs using the IVIS Spectrum System (PerkinElmer, Waltham, MA, USA). The data were analyzed by the Living Image software (PerkinElmer, Waltham, MA, USA).

### 5.17 Measurement of serum ALT, AST, IL‐6, Hyaluronic Acid, and BDCA‐3 Concentration

Mouse serum was collected 24 h after liver IRI or sham operation. The concentrations of AST, ALT, IL‐6, Hyaluronic acid, and BDCA‐3 were analyzed using the ELISA Kits for mouse AST, ALT, IL‐6, Hyaluronic acid, and BDCA‐3 in accordance with the manufacturer's instructions.

### 5.18 Flow Cytometric Analysis

Flow cytometry was used to determine the cellular uptake of NPs and phenotypes of mouse immune cells using various combinations of the following fluorescence‐labeled anti‐mouse mAbs: CD3(17A2), CD4 (GK1.5), CD8 (53‐6.7), CD11b (M1/70), CD11c (N418), CD16/32(93), CD19 (6D5), CD45 (30‐F11), F4/80 (BM8), Ly‐6G (1A8), NK1.1 (PK136). CD31(MEC13.3), CD144(BV13). Single‐cell suspensions were stained with flow cytometric antibodies for 30 min at 4°C. All samples were acquired on a BD LSRFortessa (BD Bioscience, San Jose, CA, USA). The data were analyzed by FlowJo software (Tree Star, San Carlos, CA, USA).

### 5.19 Statistical Analysis

Data are shown as mean ± SEM. All statistical analyses were performed using GraphPad Prism 8.0.1 software. Unpaired Student's *t* tests or one‐way ANOVA followed by Tukey's post hoc test were used to compare paired and unpaired data. Two‐way ANOVA followed by Tukey's post hoc test was used to analyze the effects of drugs and time on the in vivo metabolism of TSP‐1. RNA sequencing data analysis determines statistical significance through hypergeometric test and corrects p‐values for multiple testing. A p value less than 0.05 was considered statistically significant.

## Author Contributions

W.H. designed, executed, and analyzed most of the research data and helped incorporate all authors' comments into the manuscript. M.X. assisted with the experimental design. M.X., M.K., C.X., and T.H. assisted with flow cytometry studies. X.Y., Y.M., and M.C. assisted with the construction of animal models. Z.M., C.S., and H.Y. assisted with the characterization of the nanoparticles. T.S. and Y.‐G.Y. conceptualized, designed, and supervised all studies and participated in the drafting and editing of the paper. All authors critically reviewed the paper.

## Funding

This work was supported by the National Key Research and Development Program of China (2024YFA0918600). This work was supported by the National Natural Science Foundation of China (823250290, U22A20156, W2441022, 82300854). This work was supported by the Fundamental Research Funds for the Central Universities, Jilin University (China).

## Conflicts of Interest

The authors declare no conflicts of interest.

## Supporting information




**Supporting File**: advs75344‐sup‐0001‐SuppMat.docx.

## Data Availability

The data that support the findings of this study are available from the corresponding author upon reasonable request.
