# Supplementary material for: A Biomimetic Nanoparticle System Intercepts and Degrades Thrombospondin‐1 to Restore Vascular Homeostasis After Ischemic Injury
Source: Adv Sci (Weinh). 2026 Apr 20;13(38):e75344. doi: 10.1002/advs.75344 (PMC13335591; doi:10.1002/advs.75344)
Supplement: Supplementary file 1 — Supporting File: advs75344‐sup‐0001‐SuppMat.docx. [file ADVS-13-e75344-s001.docx]

Supporting Information

A Biomimetic Nanoparticle System Intercepts and Degrades Thrombospondin-1 to Restore Vascular Homeostasis after Ischemic Injury

*Haorui Wang^1,2,3^, Xiandi Meng^1,2^, Yanbao Xin^1,2^, Kuirong Mao^1,2^, Huizhu Tan^1,2^, Changkai Ma^1,2^, Xiuxiu Cong^1,2^, Mengfei Zhao^1,2^, Meiling Yu^1,2^, Si Chen^1,2^, Yue Hou^1,2^, Yong-Guang Yang^1,2,3,5 *^,* *Tianmeng Sun^1,2,3,4,5^* *^*^*

**This file includes:**

**Figure S1-28 & Table S1**


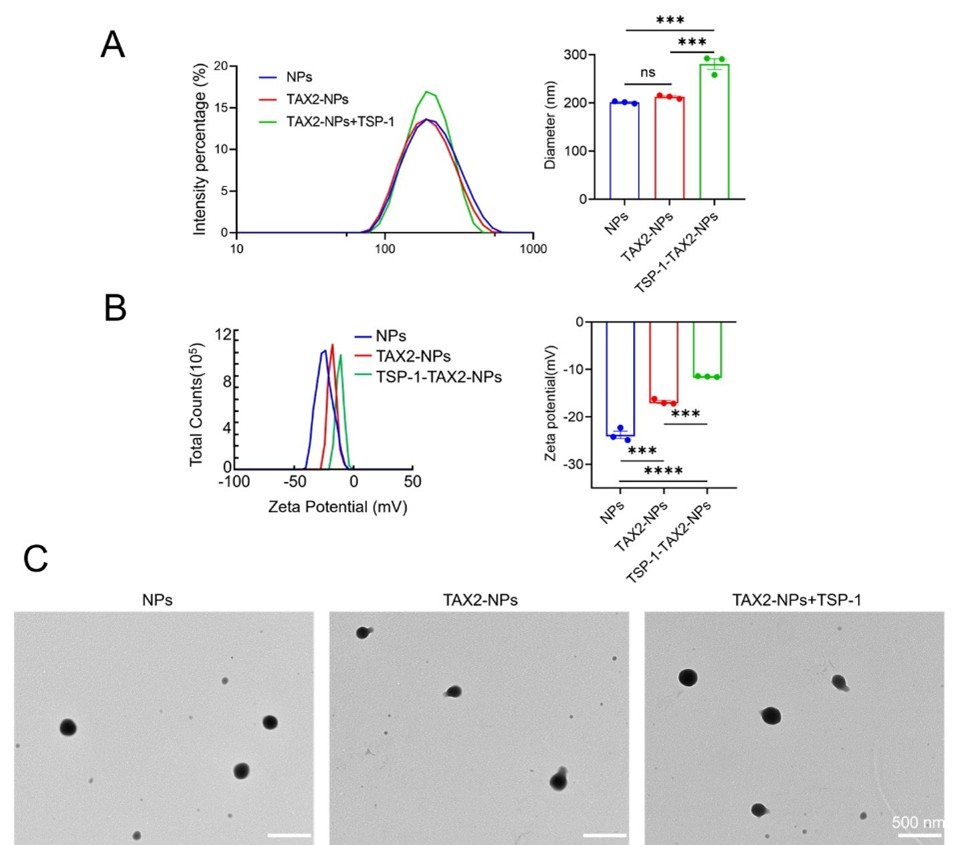


**Figure S1|** (**A**) Average particle size and size distribution of bare nanoparticles (NPs), TAX2‑modified nanoparticles (TAX2‑NPs), and TSP‑1‑bound TAX2‑NPs (TSP‑1‑TAX2‑NPs). (mean ± SEM; n = 3 independent preparations). (**B**) Zeta potential of NPs, TAX2-NPs and TSP-1-TAX2-NPs. (mean ± SEM; n = 3 independent preparations). (**C**) Representative transmission electron microscopy (TEM) images of NPs, TAX2‑NPs and TSP‑1‑TAX2‑NPs. Scale bar: 500 nm.


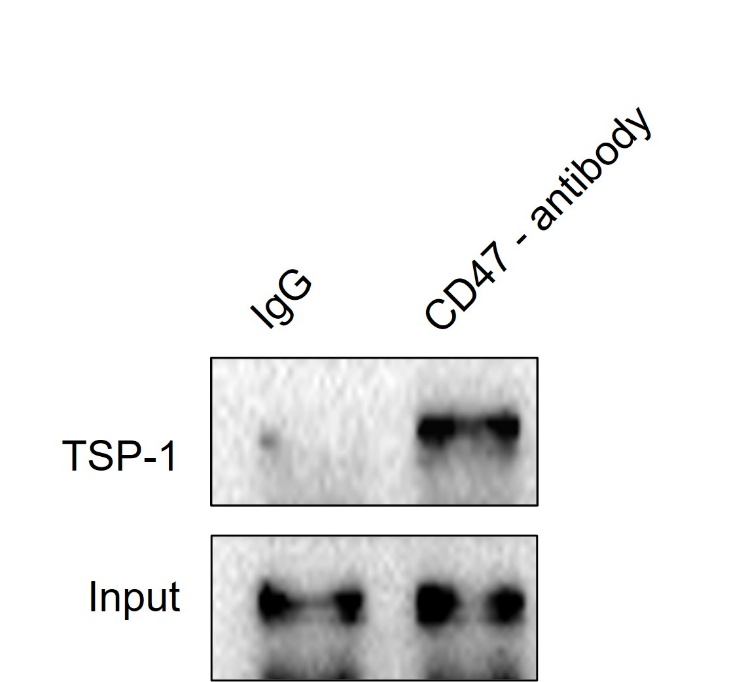


**Figure S2|** Representative Western blot images showing IgG antibody magnetic beads and CD47 antibody magnetic beads were respectively incubated with PBS whole cell lysate. The TSP-1 adsorbed by the magnetic beads was then analyzed.


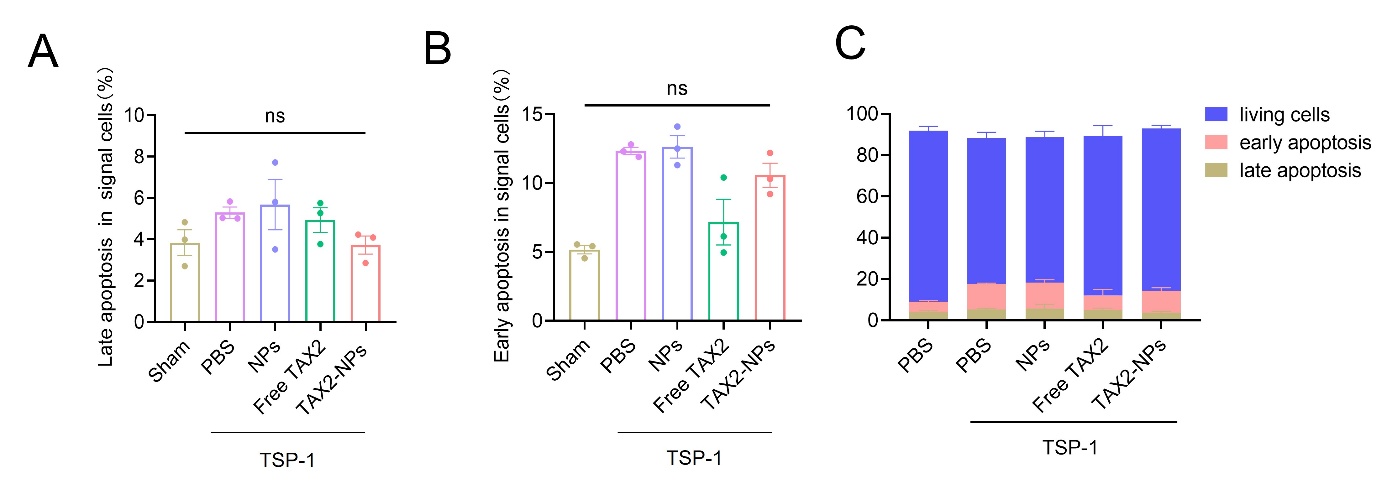


**Figure S3| (A**) Quantification of late apoptotic LSECs (mean ± SEM; n = 3 independent experiments). (**B)** Quantification of early apoptotic LSECs (mean ± SEM; n = 3 independent experiments). All experiments were analyzed using unpaired two-tailed *t*-tests. (**C)** Quantification of apoptotic and viable LSECs (mean ± SEM; n = 3 independent experiments). All experiments were analyzed using one-way ANOVA followed by Tukey’s post hoc test.


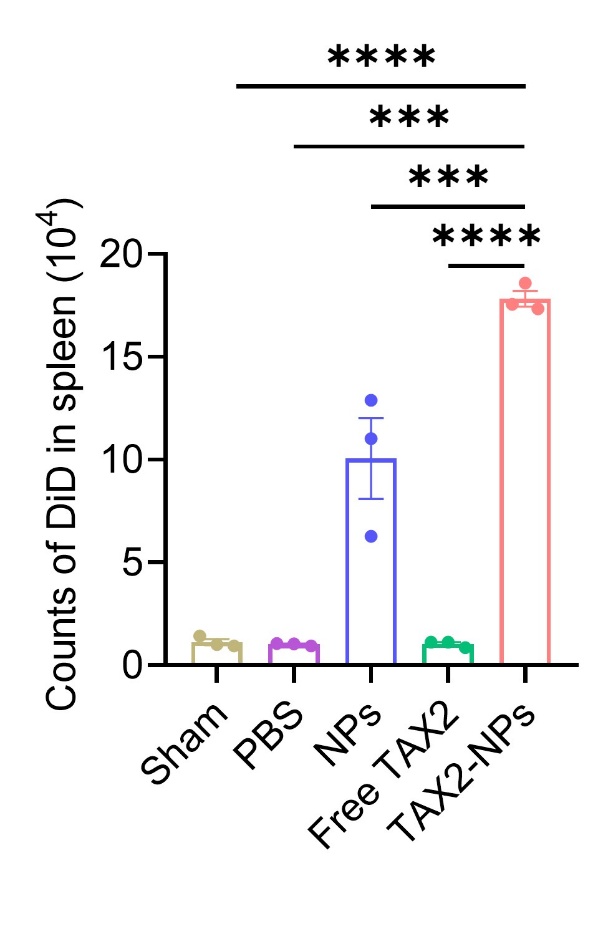


**Figure S4|** Quantification of DiD fluorescent dye in the spleen (n = 3 independent preparations).


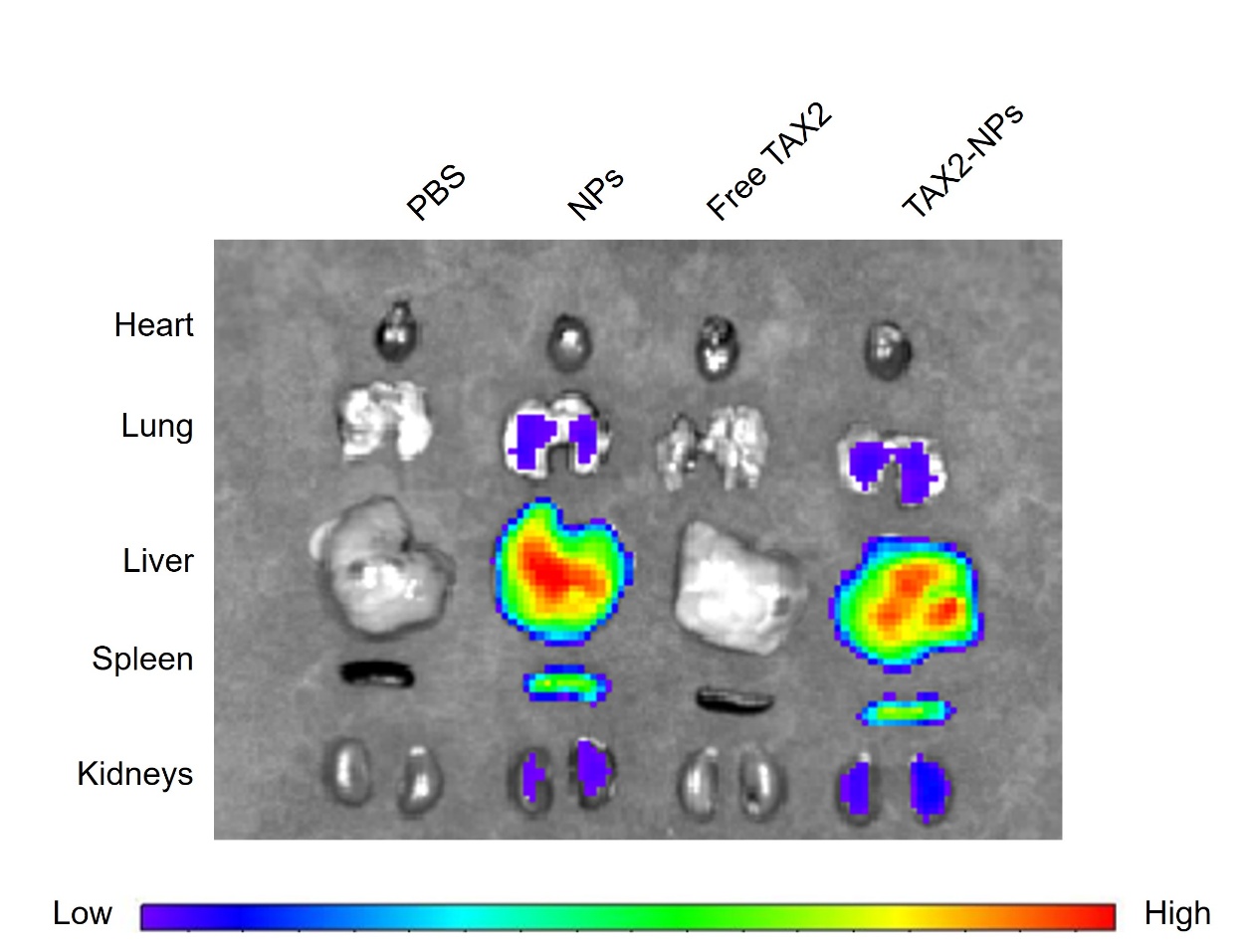


**Figure S5|** *In vivo* imaging (IVIS) of the accumulation of nanoparticles in the main organs (heart, lung, liver, spleen and kidney) of normal mice 2 hours after intravenous injection.


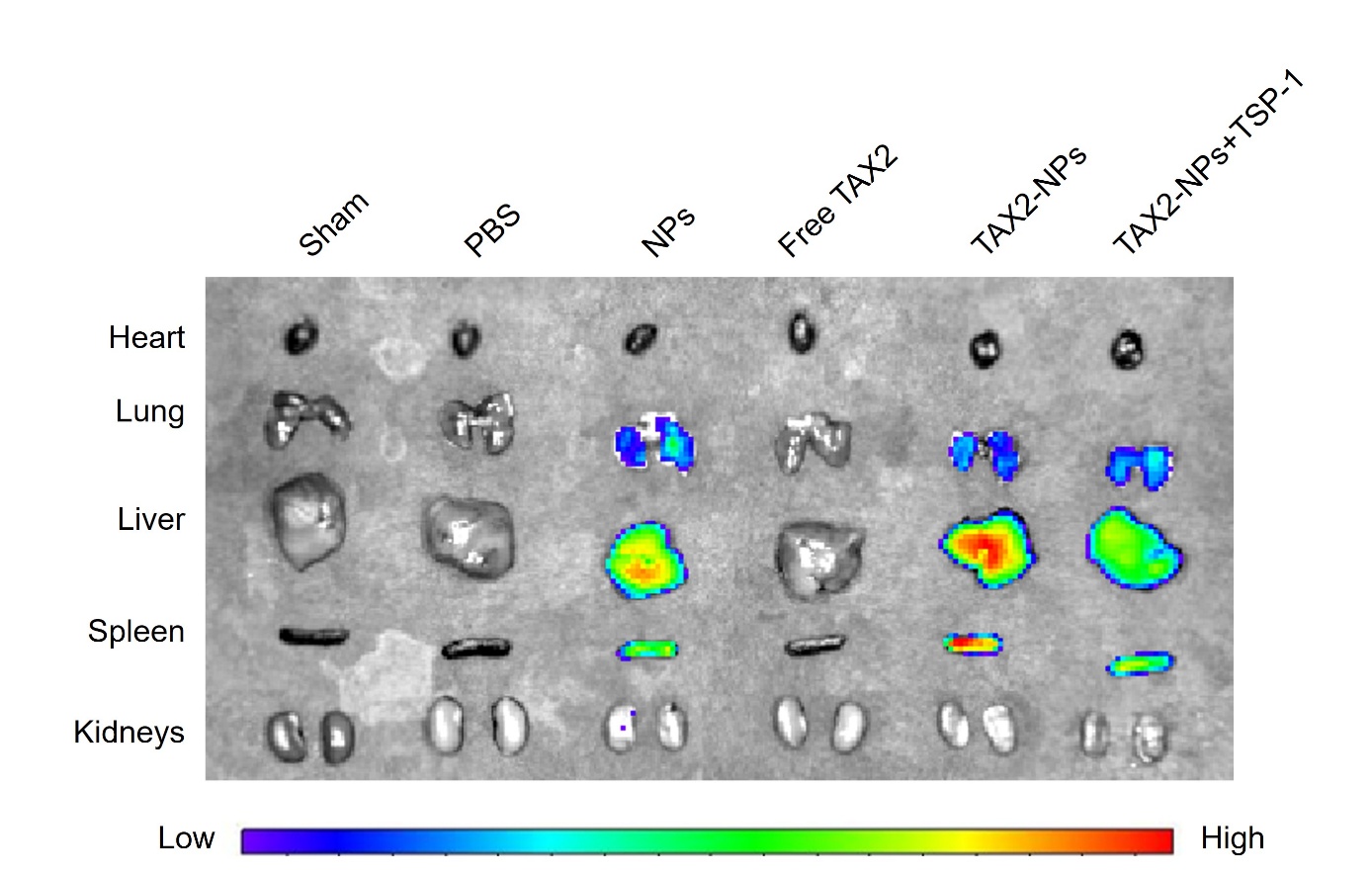


**Figure S6|** *In vivo* imaging (IVIS) of the accumulation of nanoparticles in the main organs (heart, lung, liver, spleen and kidney) of HIRI mice 2 hours after intravenous injection.


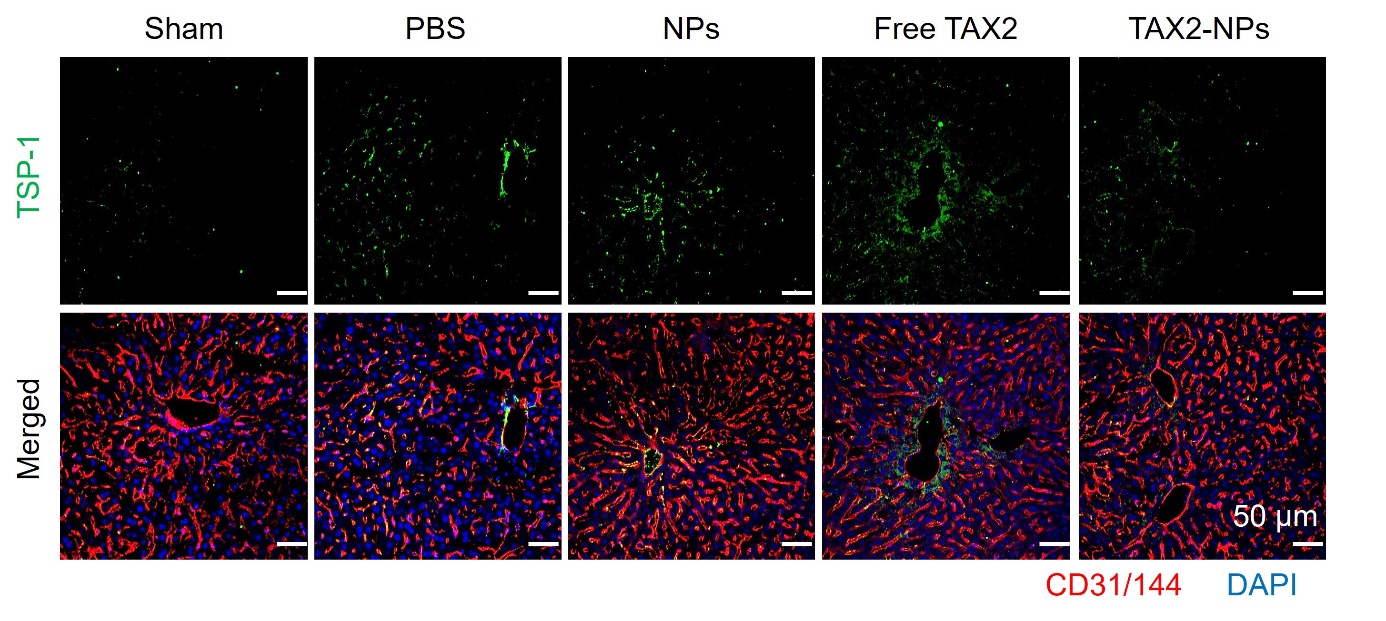


**Figure S7|** Confocal microscopy detection of co-localization of liver endothelial cells with TSP-1. (Green: TSP-1, Red: CD31/144, Blue: DAPI). scale bar 50 µm

**
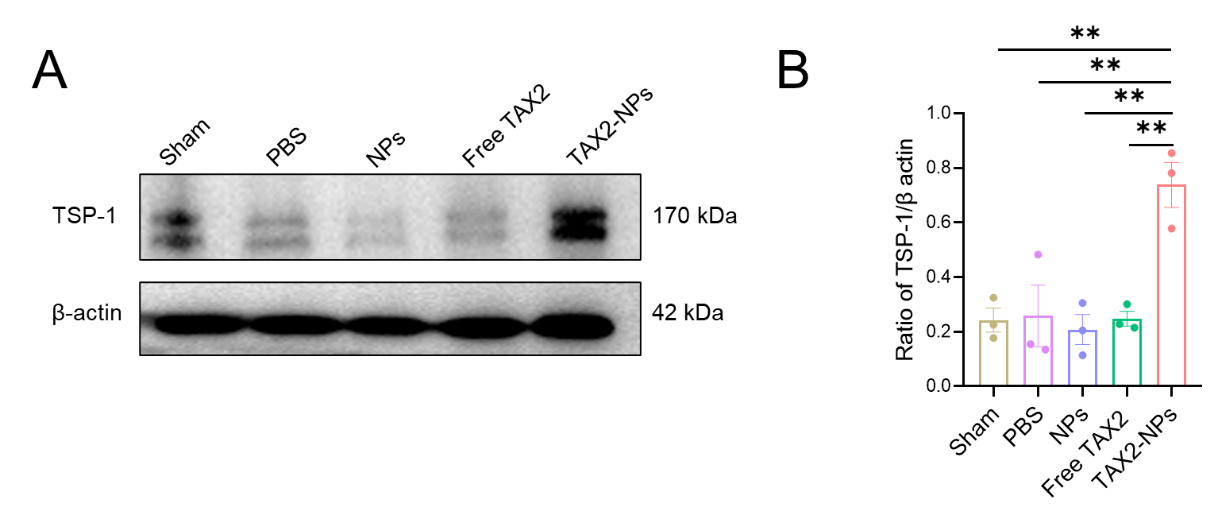
**

**Figure S8|** (**A**) Representative Western blot images of TSP-1 levels in the spleens of mice 3 hours after liver IRI in each group. (**B**) Quantification of TSP-1 levels in each group (n = 3 independently prepared). *P < 0.05, **P < 0.01, ***P < 0.001, ****P < 0.0001 by one-way ANOVA followed by Tukey’s post hoc test.


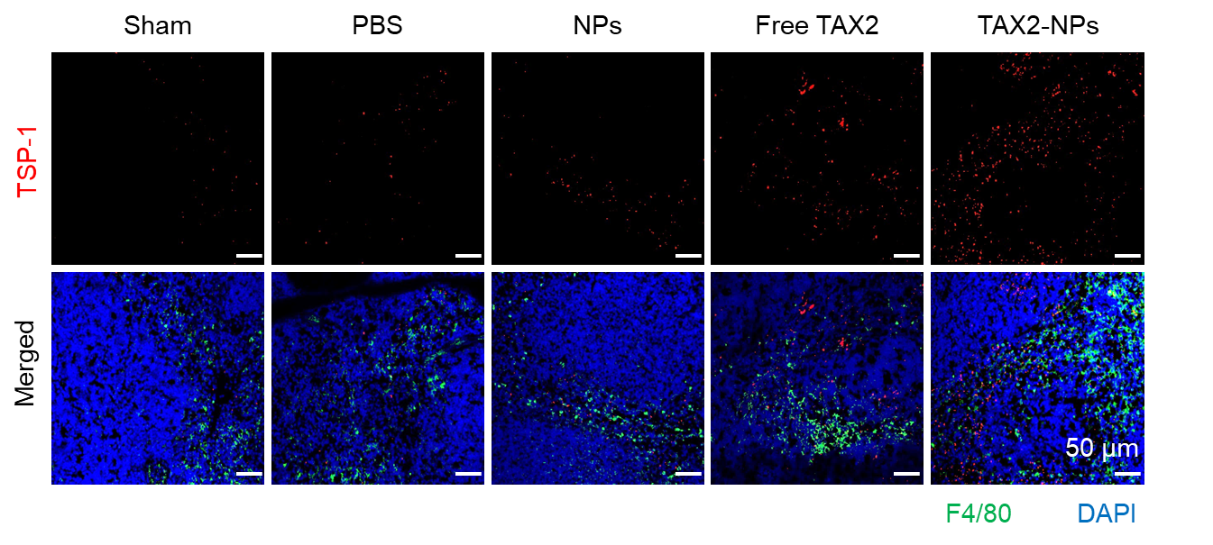


**Figure S9|** Representative images of co-localization of TSP-1 and F4/80-labeled macrophages in the spleens of each group 3 hours after liver IRI, captured by confocal microscopy, scale bar, 50 µm, (Green: F4/80, Red: TSP-1, Blue: DAPI).


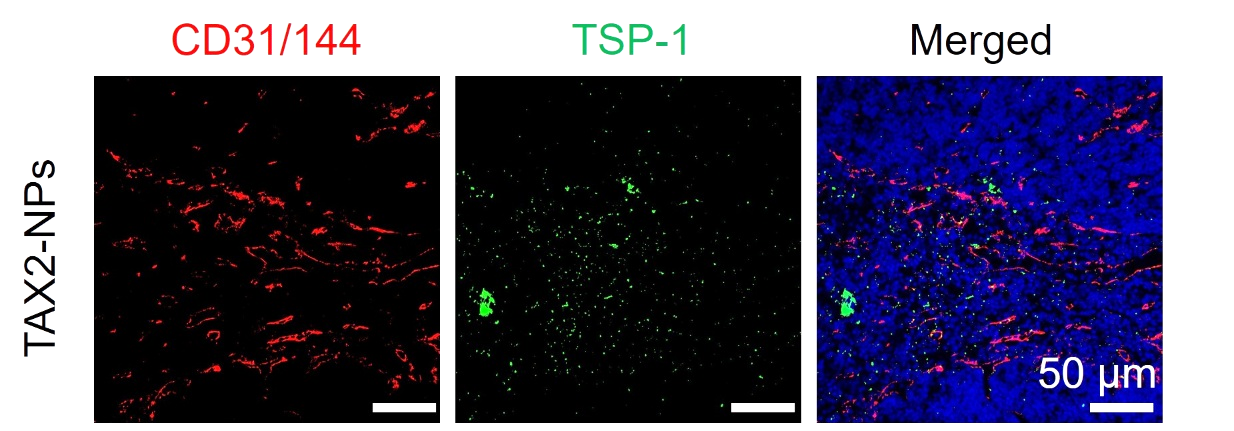


**Figure S10|** Representative images of co-localization of TSP-1 and endothelial cells in the spleens of TAX2-NPs treat group 3 hours after liver IRI captured by confocal microscopy, scale bar, 50 µm, (Green: TSP-1, Red: CD31/144, Blue: DAPI).

**
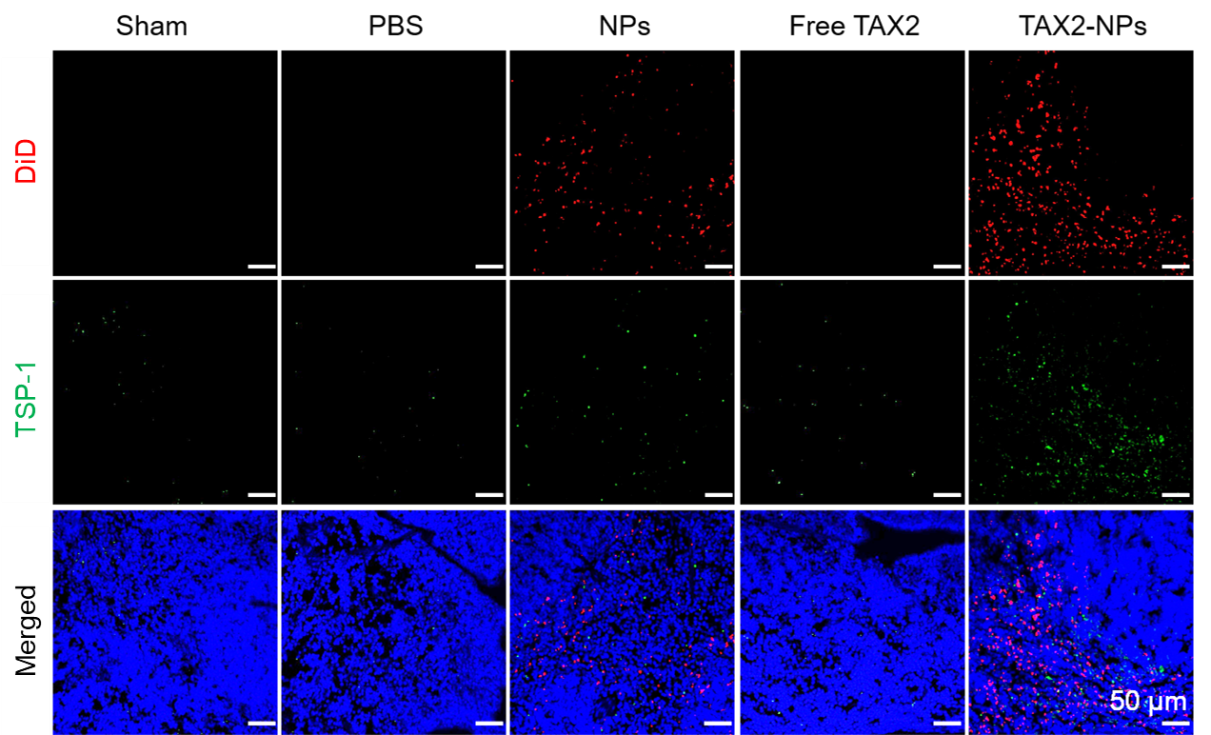
**

**Figure S11|** Representative images of co-localization of TSP-1 and DiD-labeled nanoparticles in the spleens of each group 3 hours after liver IRI captured by confocal microscopy, scale bar, 50 µm, (Green: TSP-1, Red: DiD, Blue: DAPI).


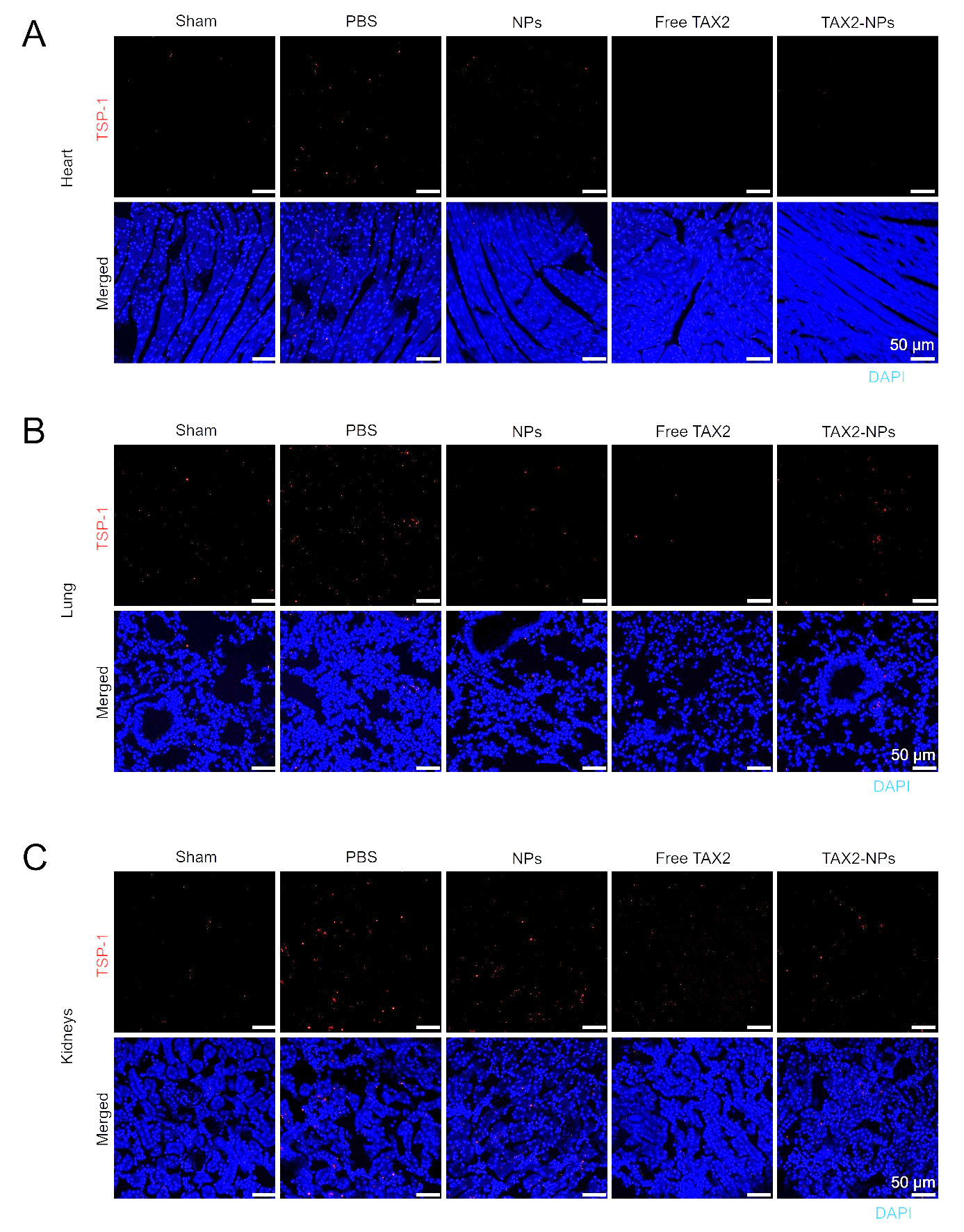


**Figure S12|** Representative images of TSP-1 in the spleens of main organs (heart, lung and kindey) of each group 3 hours after liver ischemia-reperfusion injury captured by confocal microscopy, scale bar 50 µm, (Red: TSP-1, Blue: DAPI )


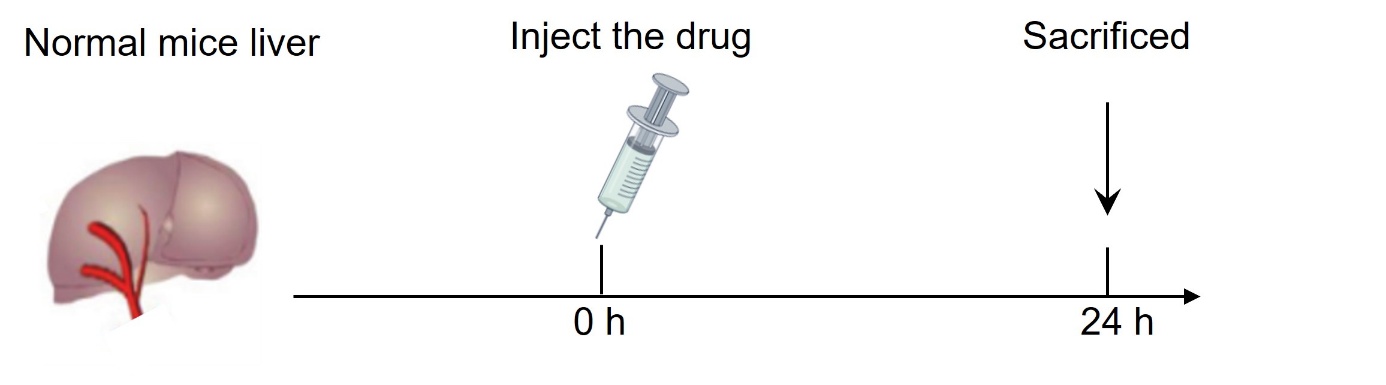


**Figure S13|** Schematic diagram of the experimental process for inducing autophagy in mouse liver by nanoparticles

Schematic diagram of the experimental process for inducing autophagy in mouse liver by nanoparticles


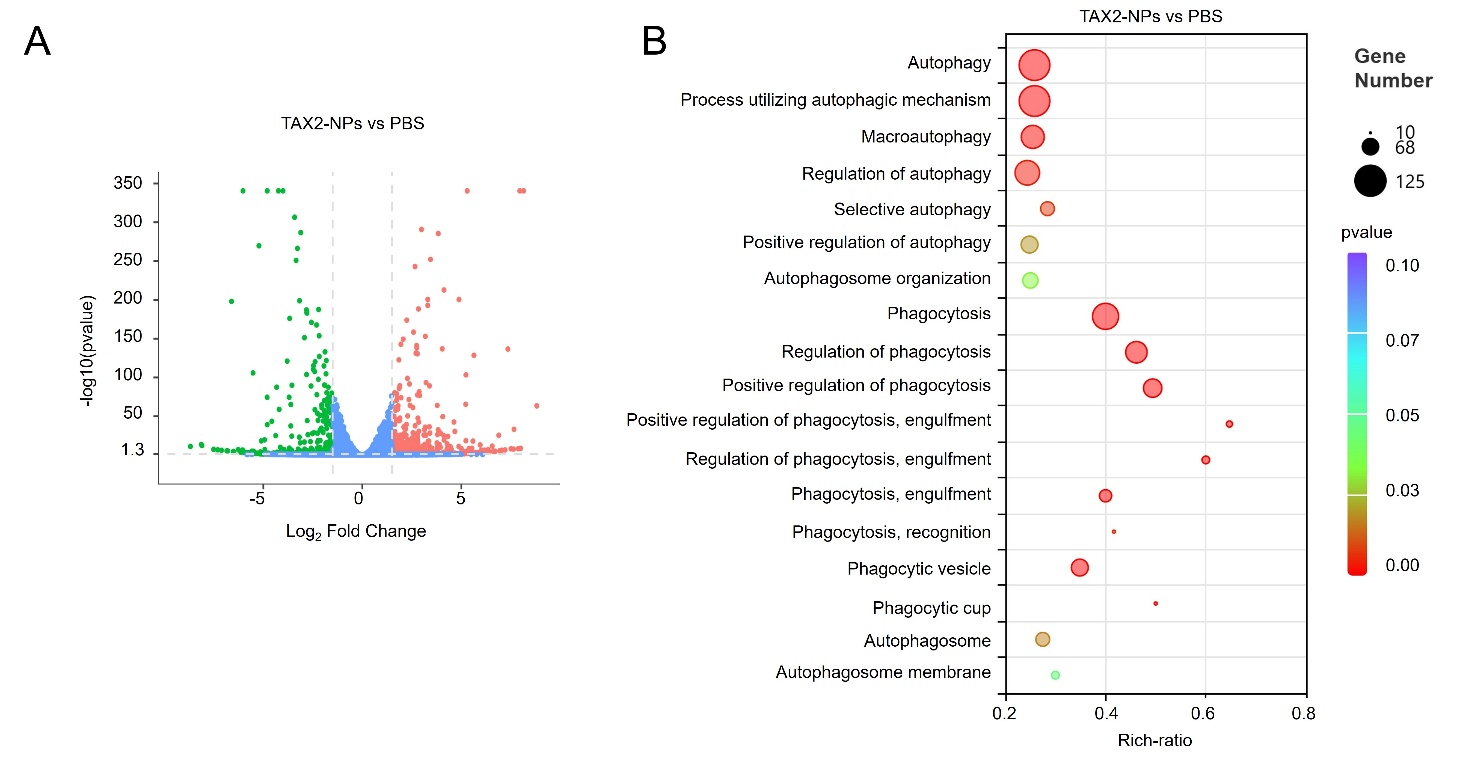


**Figure S14|** (**A**) Distribution of upregulated (720) and downregulated (395) genes in mouse liver after treatment with PBS and TAX2-NPs compared with the PBS group (|log2FC| ≥ 1.5, P value < 0.05, n=3). (**B**) GO enrichment analysis of autophagy-related signaling pathways in the upregulated gene expression in the liver tissues of mice treated with TAX2-NPs compared to those treated with PBS (p value < 0.05, n = 3 independently prepared).


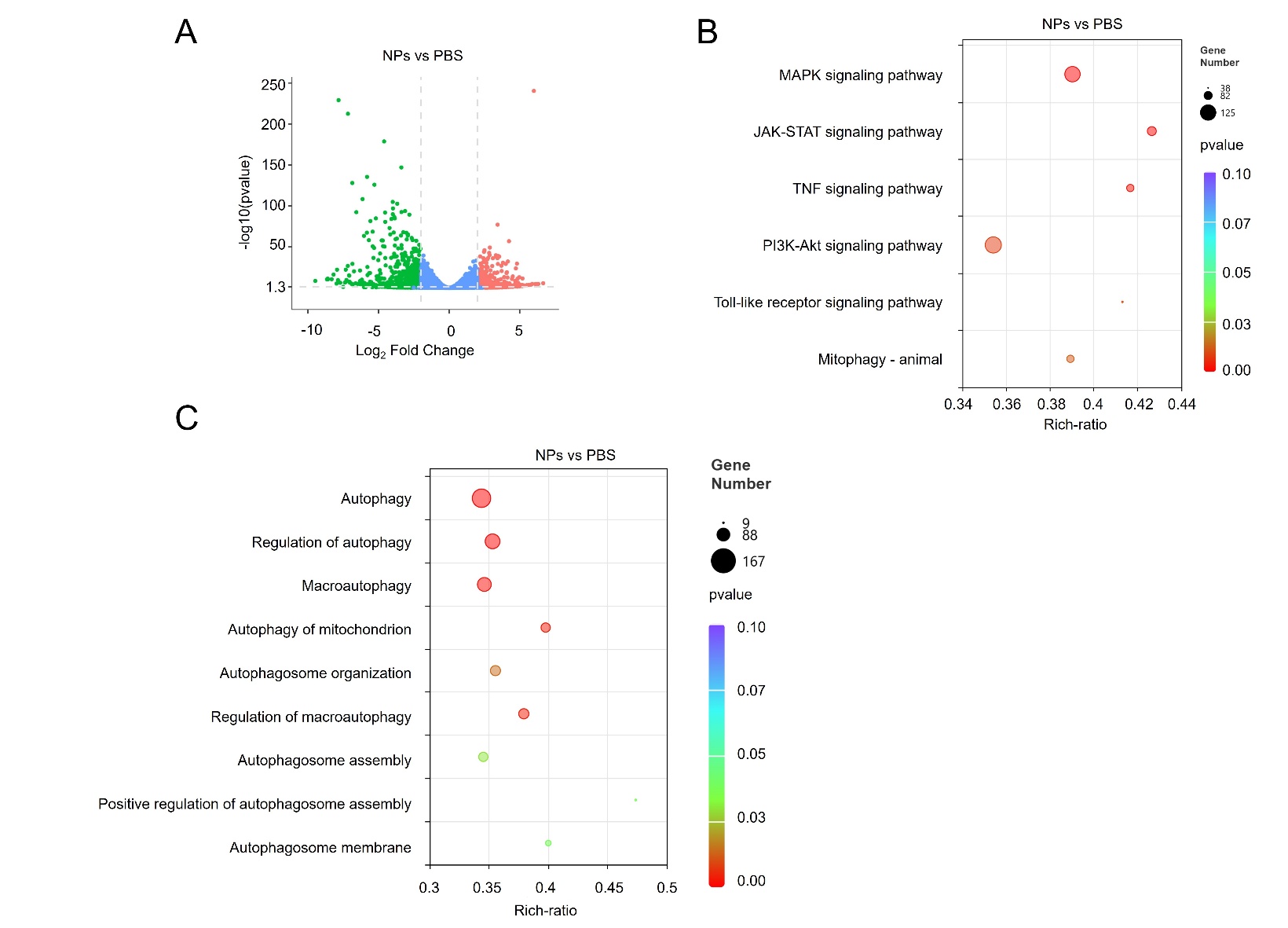


**Figure S15|** **| (A**) Distribution of upregulated (1287) and downregulated (1710) genes in mouse liver after treatment with PBS and NPs compared with the PBS group (|log2FC| ≥ 1.5, p value < 0.05, n=3 independent preparations). (**B**) KEGG enrichment analysis of autophagy-related signaling pathways in the upregulated gene expression in the liver tissues of mice treated with NPs compared to those treated with PBS (n = 3). (**C**) GO enrichment analysis of autophagy-related signaling pathways in the upregulated gene expression in the liver tissues of mice treated with NPs compared to those treated with PBS (n = 3).


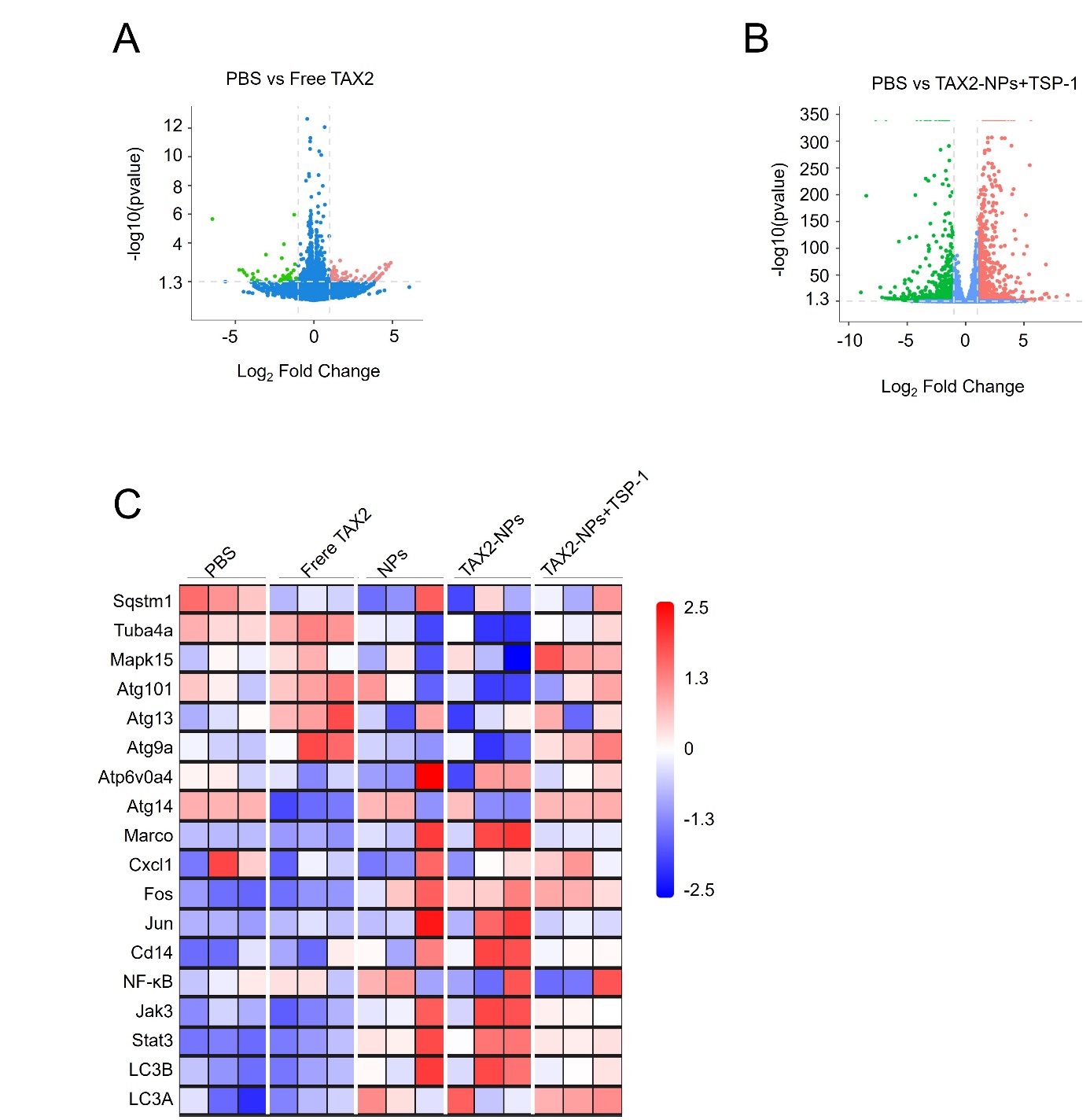


**Figure S16**| (**A**) Distribution of upregulated (72) and downregulated (44) genes in mouse liver after treatment Free TAX2 compared with the PBS group (|log2FC| ≥ 1.5, p value < 0.05, n=3 independent preparations). (**B**) Distribution of upregulated (1232) and downregulated (958) genes in mouse liver after treatment with TSP-1-TAX2-NPs compared with the PBS group (|log2FC| ≥ 1.5, p value < 0.05, n=3 independent preparations). (**C**) Heatmap of autophagy-related genes in liver tissues of normal mice treated with PBS, Free TAX2, NPs, TAX2-NPs and TSP-1-TAX2-NPs for 24 hours detected by RNA-seq (n=3 independent preparations).

**Figure S17**| Quantitative analysis of the diameter of autophagosomes produced by mouse BMDM treated with PBS, blank nanoparticles and TAX2-NPs for 24 hours as detected by transmission electron microscopy (TEM) (n = 6 independent preparations). *P < 0.05, **P < 0.01, ***P < 0.001, ****P < 0.0001 by one-way ANOVA followed by Tukey’s post hoc test


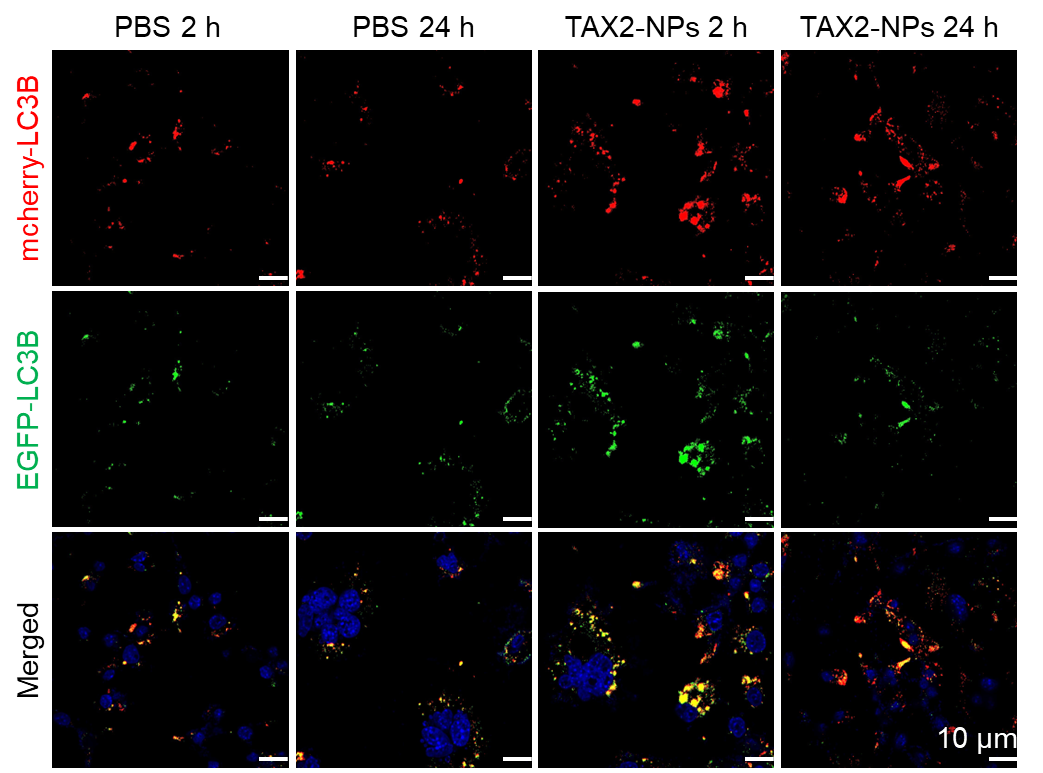


**Figure S18|** Representative images of RAW264.7 cells infected with EGFP-mCherry-LC3B virus and treated with PBS or TAX2-NPs at different time points, captured by confocal microscopy, scale bar, 10 µm, (Green: EGFP-LC3B, Red: mCherry-LC3B, Blue: DAPI).


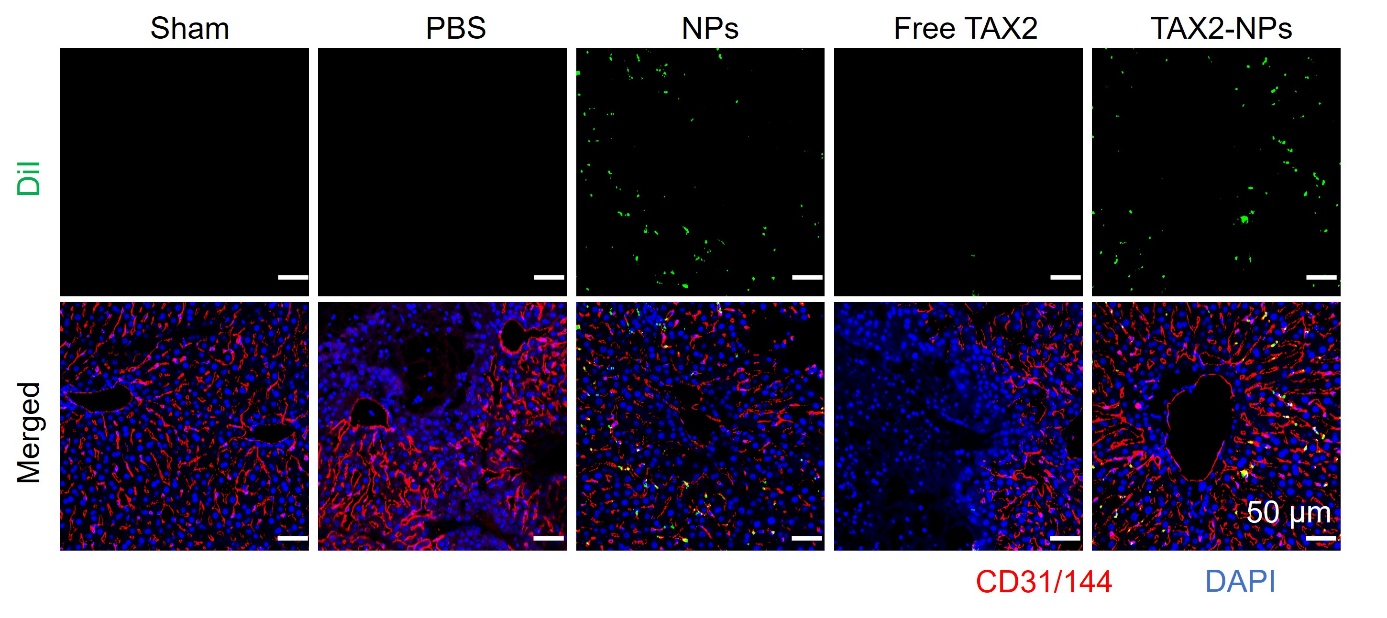


**Figure S19|** Confocal microscopy detection of co-localization of liver endothelial cells with Dil-labeled nanoparticles. (Green: Dil, Red: CD31/144, Blue: DAPI). scale bar 50 µm.


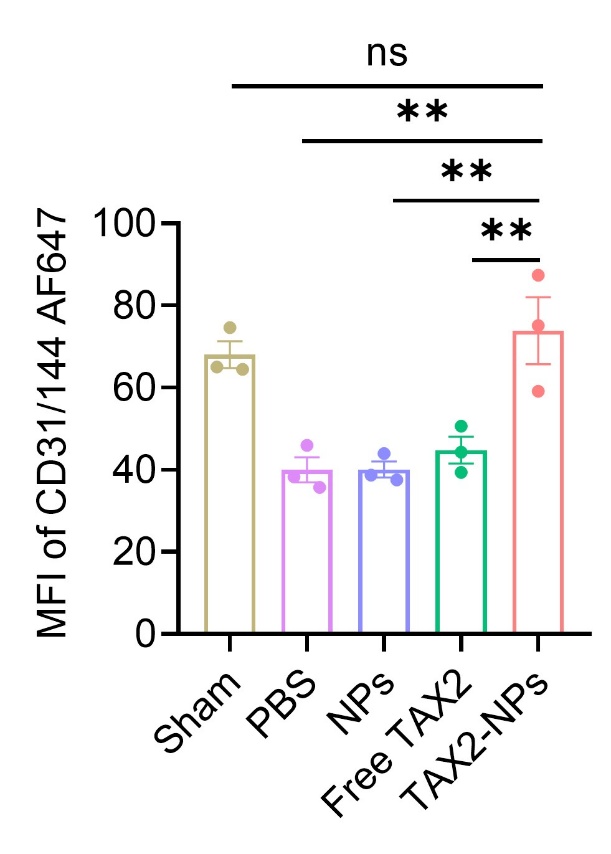


**Figure S20|** Quantitative analysis of the mean fluorescence intensity of CD31/144 (n = 3 independent preparations). *P < 0.05, **P < 0.01, ***P < 0.001, ****P < 0.0001 by one-way ANOVA followed by Tukey’s post hoc test


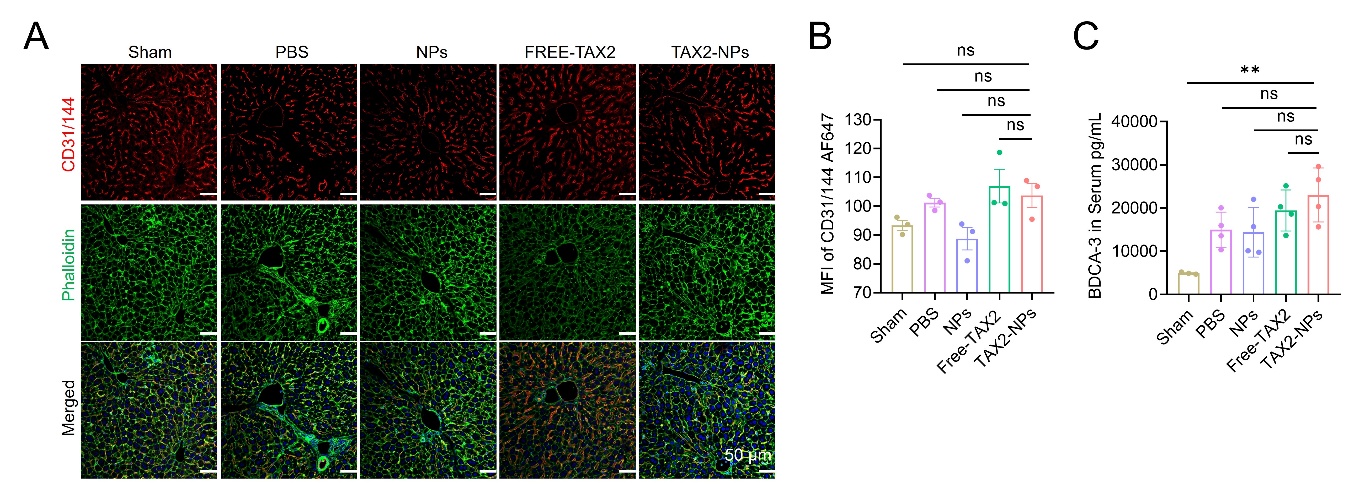


**Figure S21|** (**A**) Representative images of CD31/144 staining in the liver of TSP-1- mice 24 hours after IRI captured by confocal microscopy, scale bar, 50 µm. (Green: Phalloidin, Red: CD31/144, Blue: DAPI). (**B**) Quantitative analysis of the mean fluorescence intensity of CD31/144 (n = 3 independent preparations). (**C**) ELISA detection of BDCA-3 content in serum (n = 4 independent preparations).


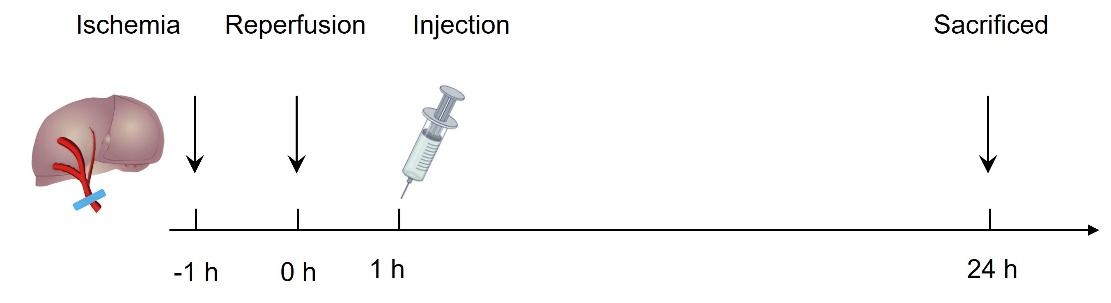


**Figure S22|** Schematic diagram of the treatment of IRI-induced apoptosis of murine hepatocytes.


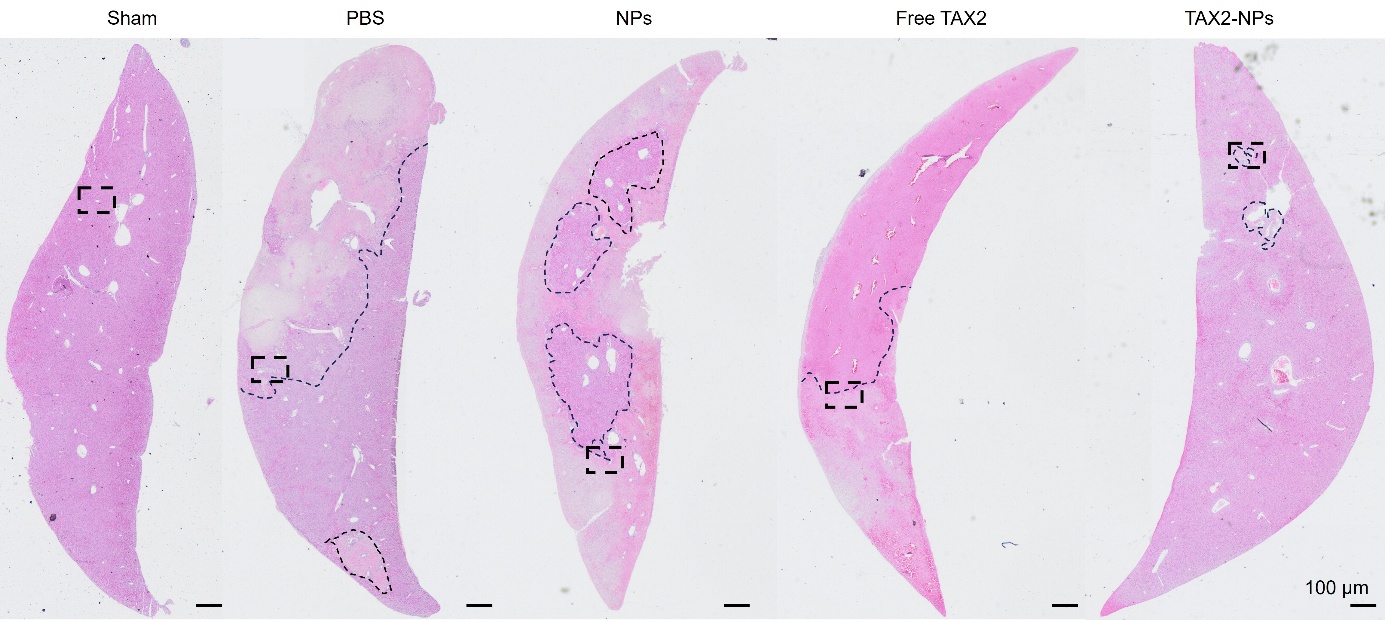


**Figure S23|** Representative full‑field images of H&E staining of IRI liver in mice, scale bar, 100 µm. The red dashed box indicates the position of the high-magnification image.


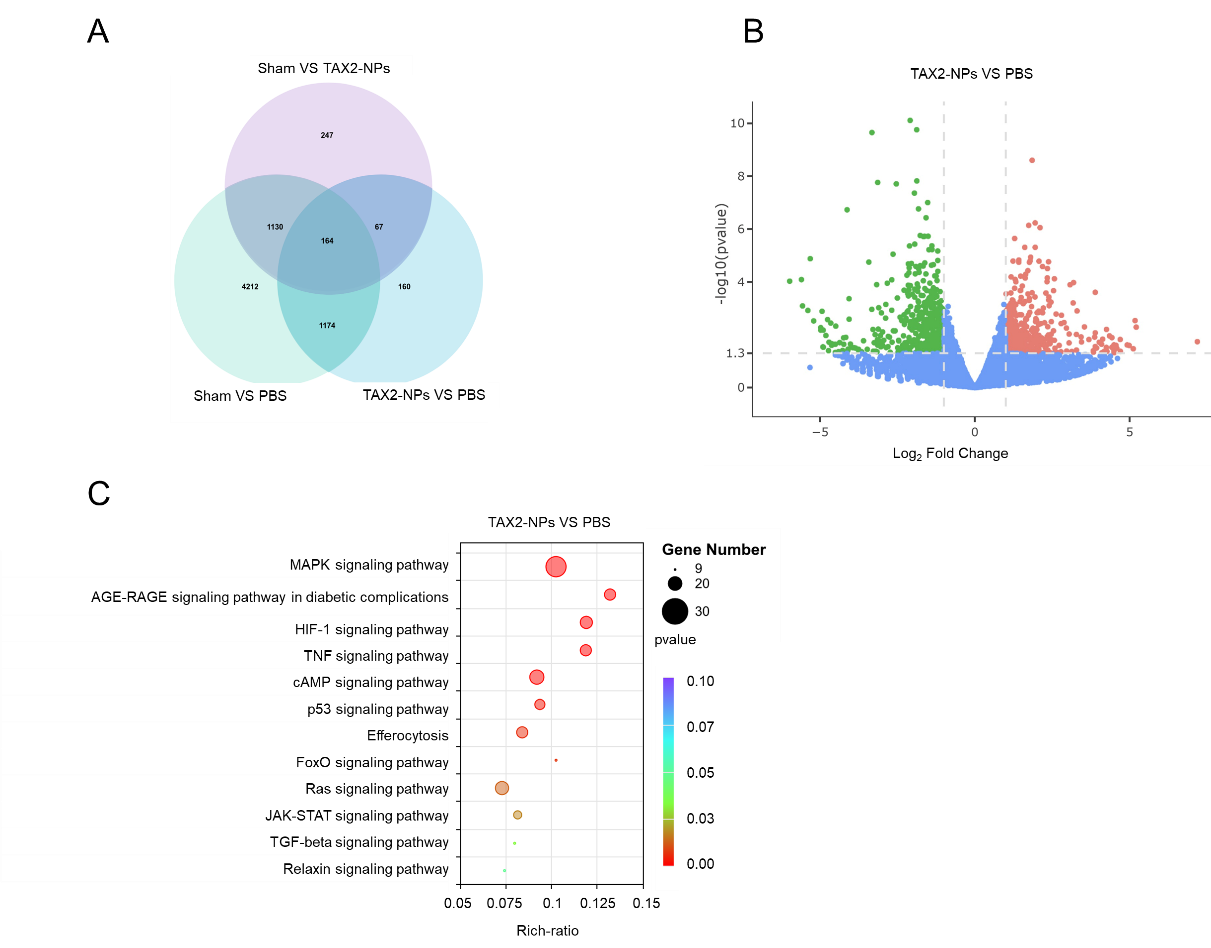


**Figure S24**| (**A**) Venn diagram showing the overlap analysis of down-regulated genes in the livers of mice in three comparisons (TAX2-NPs vs PBS, Sham operation vs TAX2-NPs, and Sham operation vs PBS). Down-regulated genes were defined as those with |log2FC| > 1 and p < 0.05 (n = 3 independent preparations). (**B**) Compared with the PBS group, the distribution of upregulated genes (463) and downregulated genes (493) in the livers of IRI mice treated with PBS and TAX2-NPs (|log2FC| > 1.0, p < 0.05, n = 3 independent samples). (**C**) KEGG enrichment analysis of down-regulated genes in the liver tissues of mice treated with TAX2-NPs compared to those treated with PBS, focusing on inflammation and apoptosis-related signaling pathways (n = 3 independent preparations).

**Figure S25**| Quantitative analysis of the mean fluorescence intensity of HNE (n = 3 independent preparations). *P < 0.05, **P < 0.01, ***P < 0.001, ****P < 0.0001 by one-way ANOVA followed by Tukey’s post hoc test

**Figure S26|** Quantitative analysis of TUNEL-positive regions (n = 3 independent preparations). *P < 0.05, **P < 0.01, ***P < 0.001, ****P < 0.0001 by one-way ANOVA followed by Tukey’s post hoc test


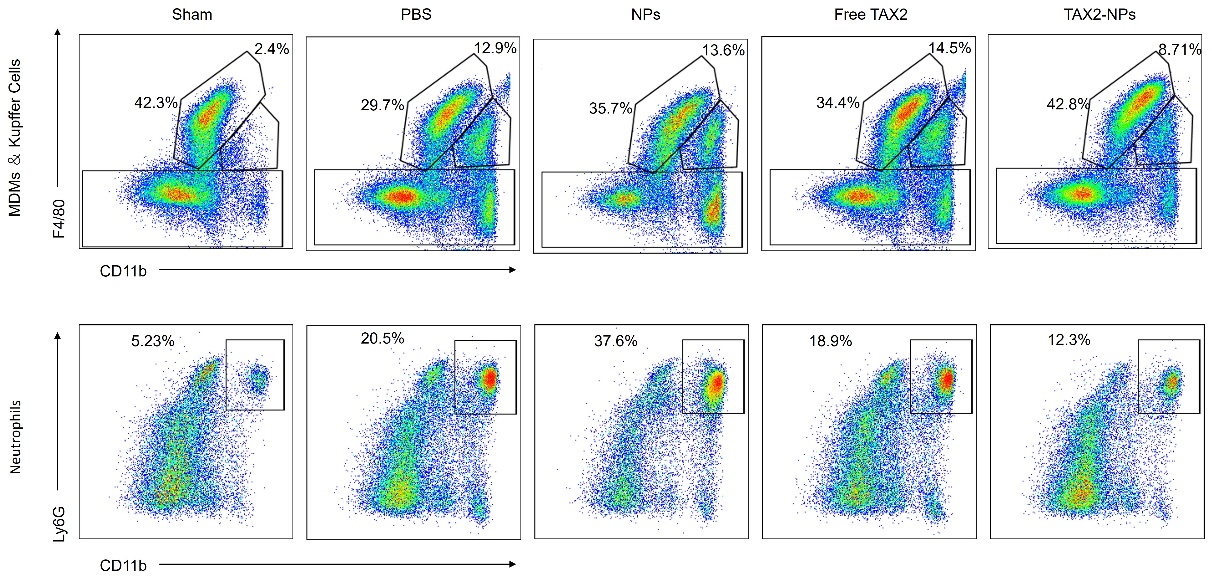


**Figure S27|** Flow Cytometry gating diagrams of Kupffer cells (CD11b⁺, F4/80^hi^), monocyte-derived macrophage (CD11b⁺, F4/80^med^), and neutrophils (CD11b^+^, Ly6G^+^, F4/80^low^, Ly6C^med^) in mouse liver.


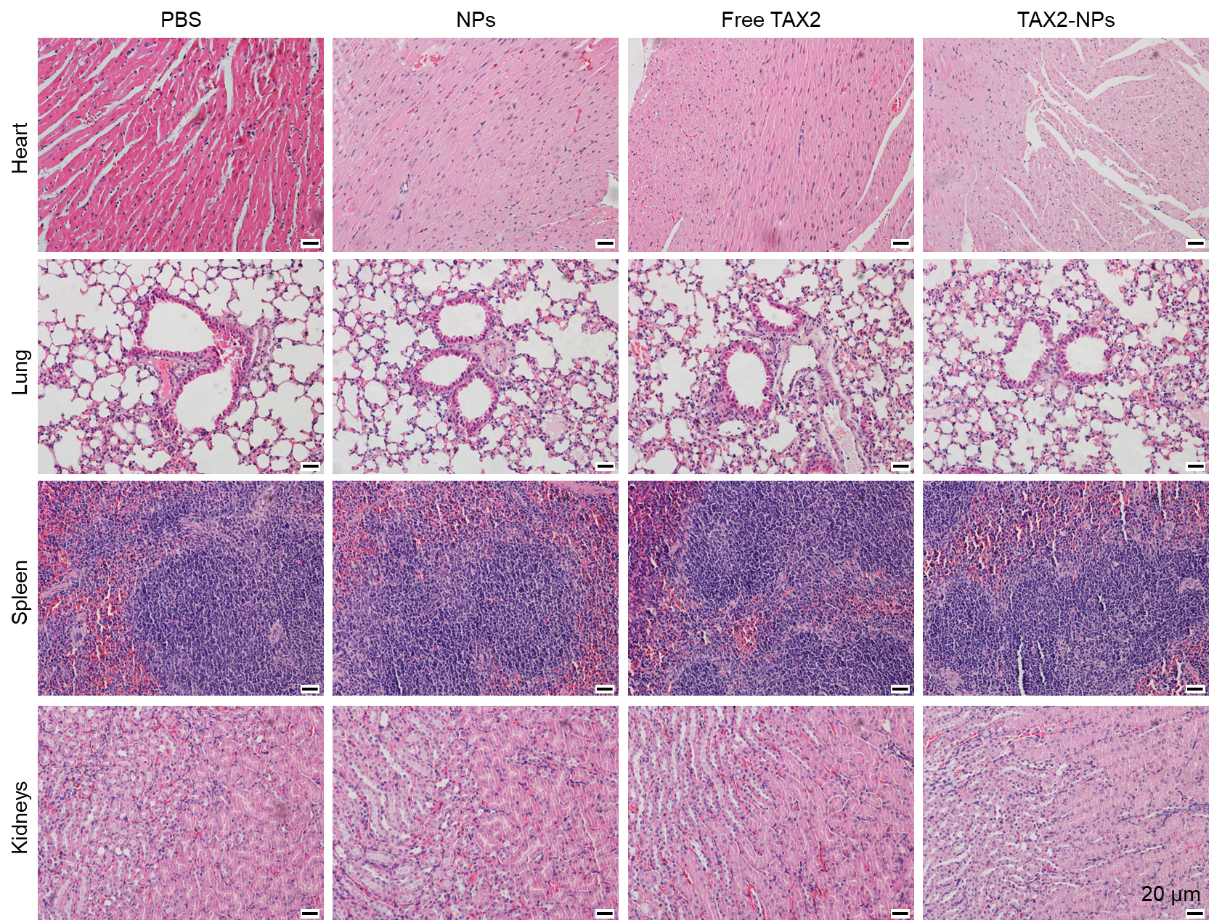


**Figure S28|** Representative H&E-stained images of the major organs including heart, lung, spleen and kidney of liver IRI mice 24 hours after injection of the designated drugs in each group. scale bar 20 µm.

**Supplemental Tables**

| N(site) K(M^-1^) ∆H(cal mol^-1^) ∆S(cal mol^-1^ deg^-1^) |
| --- |
| Free TAX 21.90±0.207 4.49E4±8.89E4 -5.442E4 -160 |
| NPs 2.21E-6±5.538 3.47E3±8.12E3 -2.221E8 -7.40E5 |
| TAX2-NPs 1.08±0.157 1.27E4±1.52E4 -4.215E4 -122 |

**Table S1**|Isothermal titration calorimetry was used to detect the number of binding sites (N), binding constant (K), molar binding enthalpy (ΔH), and molar binding entropy (ΔS) when each group bound to TSP-1.
